# Mechanistic transmission modeling of COVID-19 on the *Diamond Princess* cruise ship demonstrates the importance of aerosol transmission

**DOI:** 10.1073/pnas.2015482118

**Published:** 2021-02-03

**Authors:** Parham Azimi, Zahra Keshavarz, Jose Guillermo Cedeno Laurent, Brent Stephens, Joseph G. Allen

**Affiliations:** ^a^Environmental Health Department, Harvard T.H. Chan School of Public Health, Boston, MA 02115;; ^b^Department of Civil, Architectural, and Environmental Engineering, Illinois Institute of Technology, Chicago, IL 60616

**Keywords:** COVID-19, transmission risk model, aerosol transmission, Diamond Princess Cruise Ship, built environment

## Abstract

Several lines of existing evidence support the possibility of airborne transmission of coronavirus disease 2019 (COVID-19). However, quantitative information on the relative importance of transmission pathways of severe acute respiratory syndrome coronavirus 2 (SARS-CoV-2) remains limited. To evaluate the relative importance of multiple transmission routes for SARS-CoV-2, we developed a modeling framework and leveraged detailed information available from the *Diamond Princess* cruise ship outbreak that occurred in early 2020. We modeled 21,600 scenarios to generate a matrix of solutions across a full range of assumptions for eight unknown or uncertain epidemic and mechanistic transmission factors. A total of 132 model iterations met acceptability criteria (*R*^2^ > 0.95 for modeled vs. reported cumulative daily cases and *R*^2^ > 0 for daily cases). Analyzing only these successful model iterations quantifies the likely contributions of each defined mode of transmission. Mean estimates of the contributions of short-range, long-range, and fomite transmission modes to infected cases across the entire simulation period were 35%, 35%, and 30%, respectively. Mean estimates of the contributions of larger respiratory droplets and smaller respiratory aerosols were 41% and 59%, respectively. Our results demonstrate that aerosol inhalation was likely the dominant contributor to COVID-19 transmission among the passengers, even considering a conservative assumption of high ventilation rates and no air recirculation conditions for the cruise ship. Moreover, close-range and long-range transmission likely contributed similarly to disease progression aboard the ship, with fomite transmission playing a smaller role. The passenger quarantine also affected the importance of each mode, demonstrating the impacts of the interventions.

## Significance

We find that airborne transmission likely accounted for >50% of disease transmission on the *Diamond Princess* cruise ship, which includes inhalation of aerosols during close contact as well as longer range. These findings underscore the importance of implementing public health measures that target the control of inhalation of aerosols in addition to ongoing measures targeting control of large-droplet and fomite transmission, not only aboard cruise ships but in other indoor environments as well. Guidance from health organizations should include a greater emphasis on controls for reducing spread by airborne transmission. Last, although our work is based on a cruise ship outbreak of COVID-19, the model approach can be applied to other indoor environments and other infectious diseases.

Understanding the importance of each transmission pathway for COVID-19 is critical to informing public health guidelines for effectively managing the spread of the disease. Although information and guidance on the likely routes of transmission of severe acute respiratory syndrome coronavirus 2 (SARS-CoV-2) continue to evolve, quantitative information on the relative importance of specific transmission pathways remains limited (1). The current position of the World Health Organization (WHO) is that the COVID-19 virus is transmitted primarily through respiratory droplets (assumed >5 to 10 µm in diameter) and direct and indirect contact routes, while airborne transmission of the COVID-19 virus via smaller aerosols (assumed <5 µm) is likely not a major route of transmission other than in settings in which aerosol-generating procedures are occurring (2). Similarly, the US Centers for Disease Control and Prevention (CDC) has updated their position multiple times and currently maintains that “COVID-19 is thought to spread mainly through close contact from person-to-person” (which CDC defines as within about 1.8 m) and that fomite transmission and inhalation of respiratory droplets are likely not the main ways that the virus spreads (3). CDC has also acknowledged that airborne transmission by smaller droplets traveling more than 1.8 m away from infected individual(s) can sometimes occur (4).

Since the beginning of the pandemic, numerous researchers (5–15) and professional societies [e.g., American Society of Heating, Refrigerating and Air-Conditioning Engineers (16)] have raised concerns that transmission of SARS-CoV-2 can occur from both symptomatic and asymptomatic (or presymptomatic) individuals to others beyond close-range contact through a combination of larger respiratory droplets that are carried further than 1 to 2 m via airflow patterns and smaller inhalable aerosols that can remain suspended and easily transport over longer distances. These concerns arise from a growing understanding of human respiratory emissions (17, 18), known transmission pathways of other respiratory viruses (19), recent empirical evidence detecting SARS-CoV-2 in aerosol and surface samples in health care settings (20–25), and recent case studies demonstrating the likely importance of longer-range aerosol transmission in some settings (26–28).

In the absence of empirical studies using controlled exposures to elucidate transmission pathways (29), mathematical modeling approaches can offer insights into the likely importance of the different modes of disease transmission among human populations (30–34), provided that sufficiently accurate inputs are available. To help fill these knowledge gaps, this work uses a mechanistic modeling approach to investigate the relative importance of multiple transmission routes of SARS-CoV-2 among individuals aboard the *Diamond Princess* cruise ship, which experienced a major outbreak of COVID-19 in early 2020.

## Materials and Methods

The *Diamond Princess* cruise ship presents a unique built environment case study, with a known number of passengers, crew members, and COVID-19 cases over time, discovered through high rates of testing, and a relatively high degree of knowledge of several important human and built environment factors. The *Diamond Princess* experienced a major outbreak of COVID-19 in early 2020, with 712 of 3,711 passengers and crew members on board becoming infected (19% of the community) (35) and at least 57 other passengers who tested positive in the days after they left the ship and returned to their home countries (36). As reported, the COVID-19 outbreak was traced to a single passenger from Hong Kong who boarded the ship in Yokohama on January 20 and then disembarked in Hong Kong on January 25. He had symptoms including coughing before boarding and was diagnosed with COVID-19 on February 1 in Hong Kong. The first 10 cases were confirmed on February 4 after the ship arrived in the Yokohama port. Laboratory-confirmed cases of COVID-19 led to the quarantine of passengers aboard the *Diamond Princess* for 14 d beginning on February 5 at 7 AM, with all passengers required to remain in their cabins essentially all of the time. As of February 5, there were a total of 3,711 individuals onboard the *Diamond Princess*, with 2,666 passengers and 1,045 crew members (37).

To estimate the likely contributions of specific infection transmission modes to the number of COVID-19 cases among individuals aboard the *Diamond Princess* cruise ship, a combination of epidemic, mechanistic transmission, and dose–response models was adopted. Full model details are described in *SI Appendix*. Briefly, we utilize a stochastic Markov chain process to trace close- and long-range transmission by a combination of deposition of large respiratory droplets, inhalation of smaller aerosols, and contact with fomites under a wide range of possible scenarios constructed from combinations of unknown or uncertain input parameters.

The Markov chain model then informs a dose–response model, which in turn informs an epidemic model to generate estimates of daily and cumulative daily case counts aboard the ship from January 20 (when there was only one index case aboard the ship) to February 24 (when all passengers disembarked). We analyze only those model scenarios that achieved an acceptable agreement between predicted and reported case numbers for daily cumulative cases (defined as *R*^2^ > 0.95) and daily cases (defined as nonnegative *R*^2^) to infer likely values of the unknown or uncertain model parameters and to quantify the contribution of the various modes of transmission in the most successful model scenarios.

### Markov Chain Model.

The Markov model adopted in this study uses a discrete-time discrete-space Markov chain to estimate the number of SARS-CoV-2 copies present in numerous physical states, as well as the probability of transmission of SARS-CoV-2 between each defined state, aboard the ship over time (*SI Appendix*, section 1). A Markov chain is a random process that undergoes transitions from one state to another in a state space. Physical elements (e.g., room air and surfaces, human skin and mucous membranes, etc.) and pathogen removal mechanisms (e.g., loss of viability, ventilation, and filtration) in the source environment–receptor pathways are represented as “states” in a discrete-time Markov chain model. Pathogens can be transferred and exchanged between states due to physical mechanisms such as emission, deposition, resuspension, filtration, and ventilation.

We chose the Markov Chain model over other existing infection transmission risk models because of its ability to stochastically track all modes of transmission under a wide variety of assumptions and with high computational efficiency. This approach offers advantages over the extensively used Wells–Riley model for quantitative infection risk assessment of respiratory infectious diseases in indoor environments, which does not consider all disease transmission routes (38). The latter reasoning offers advantages over infection transmission models that are based on more complex and computationally intensive computational fluid dynamics (CFD) simulations (39), as the Markov chain model does not require solving the partial differential equations for governing the particle transport. Moreover, others have shown that the Markov chain model can predict transient particle transport in enclosed environments with similar accuracy to Eulerian and Lagrangian models, which have been widely used for particle modeling in CFD tools (40). Many scientists also have used Markov model for predicting the transmission risk of infectious disease indoors (33, 41–45).

We considered the following 12 states for the Markov chain process, with state numbers corresponding to *SI Appendix*, Fig. S1: (#1 and 3) indoor air and surfaces of cabins occupied by infectors; (#2 and 4) public area indoor air and surfaces; (#5 to 7) hands (palms), upper respiratory tract (URT), and lower respiratory tracts (LRT) of uninfected individuals who were cabinmates of infected individuals before they became infected; (#8 to 10) palms, URT, and LRT of uninfected individuals who were not cabinmates of infected individuals before they became infected; (#11) heating, ventilation, and air-conditioning systems; and (#12) inactivation of viable virus. We conservatively (i.e., favoring against long-range airborne transmission) assumed cabins are positively pressurized and there was no air recirculation (*SI Appendix*, section 1.1.2); therefore, infectious particle could reach to a “clean” cabin only if an infector occupies it. We generated a new Markov chain matrix for each day in the simulation period to model mechanistic transmission and infection probability based on a number of assumptions for built environment parameters, crew and passengers’ interactions, adopted infection control strategies, and the number of infectors and susceptible individuals estimated from application of the transmission risk model to the previous days. The model then adjusts the number of cabins with infected individuals present at the end of each simulation day based on the number of new infected cases stemming from interactions in the common areas.

The modeling framework incorporates available empirical data on key mechanisms of SARS-CoV-2 dynamics culled from recent literature, including 1) viral RNA emission rates in large droplets (assumed greater than ∼10 µm, consistent with the conventional WHO definition) and inhalable aerosols (assumed less than ∼10 µm) from infected individuals, which were back-calculated from recent reports of air and surface sampling in health care settings and were assumed to be the same ratio for all infected individuals; 2) viability loss in air and on surfaces reported in controlled studies; and 3) estimates of aerosol deposition rates to surfaces based on typical assumptions for aerosol dynamics.

The framework also leverages estimates and assumptions for several human and built environment transmission factors, culled from prior literature where possible, including average rates of face and surface touching, inhalation rates, the shape and size of close-contact zones, time spent in various environments (e.g., public areas and cabins), floor areas and volumes of cabins and public areas, the probability of uninfected individuals within close proximity of an infected individual, and the impact of infection control strategies that were implemented during the quarantine period (e.g., mask wearing, hand washing, and surface disinfection). Detailed descriptions of all model inputs are provided in *SI Appendix*, section 1 (for relatively certain parameters) and *SI Appendix*, section 3 (for relatively unknown or uncertain parameters).

### Dose–Response Model.

To estimate the infection probability of SARS-CoV-2 viruses deposited to different body sites of susceptible individuals, we used a negative exponential dose–response model, which implies that a single particle can start an infection and all single particles are independent of each other. The probability of infection for one susceptible individual $\left(P_{infection}\right)$ in the cruise ship was calculated using Eq. 1:

$$P_{infection}=\frac{Number\;of\;infected\;cases}{Number\;of\;susceptibles}=1-\exp\left[-\left(\alpha_{URT}\times N_{URT}+\;\alpha_{LRT}\times N_{LRT}\right)\right],$$ [1]

where $N_{URT}$ and $N_{LRT}$ represent numbers of viable SARS-CoV-2 RNA copies in URT and LRT of one susceptible individual; and $\alpha_{URT}$ and $\alpha_{LRT}$, infectivity of SARS-CoV-2 for URT and LRT.

The 50% infectious dose $\left(ID_{50}\right)$, or the number of viruses necessary to infect a susceptible individual in 50% of a sample population, of SARS-CoV-2 for URT and LRT can be estimated from Eq. **2** (41, 46):

$$ID_{50}=\frac{\ln\left(2\right)}{\alpha },\;\mathrm{where}\;ID_{50}\geq \text{ln}\left(2\right).$$ [2]

Estimates of ID_50_ and infectivity for URT and LRT play a critical role in understanding the transmission of airborne infectious diseases. However, we are not aware of any clinical studies to date that report these values for SARS-CoV-2 in humans or animals. Moreover, the proportions of SARS-CoV-2 depositing in the LRT and URT of a susceptible individual when they inhale infectious aerosols are not yet characterized. Therefore, we tested three logarithmically spaced assumptions for the ratio of the effective ID_50_ for SARS-CoV-2 for aerosol inhalation (assuming deposition in the LRT) and fomite and droplet deposition (assuming deposition in the URT): ID_50_ URT:LRT = 1:1, 10:1, and 100:1. Our assumptions for equal or higher SARS-CoV-2 median infectious doses for fomite and droplet deposition in comparison to aerosol inhalation are generally in line with existing studies showing SARS-CoV-2 preferentially replicates deeper in the lungs (15) and leads to clinical symptoms at lower doses of aerosol exposures compared to ocular or intranasal routes in animal models such as African green monkeys (47, 48) and golden hamsters (49). We rely on our model approach to back-calculate effective ID_50_ values (using a basis of RNA copies) by analyzing successful model results, as described in *SI Appendix*, section 1.3. This approach allows us to test scenarios with these uncertain parameters without knowing (or needing to know) the actual magnitude of ID_50_, which can then be used to infer the likely magnitude of this ratio based on successful model iterations.

### Transmission Mode Contribution to Infection.

In addition to estimating the number of infected cases with the model framework, we also estimated the contribution of multiple infection transmission modes to the estimated number of infected cases in both cabins and public areas, including 1) direct deposition of respiratory droplets (within close range only), 2) contact with fomites, and 3) inhalation of aerosols (with both close- and long-range transmission traced separately) (Eq. 3):

$$C_{infection,\;k,r,p}=\sum_{l=0}^{D_{p}}\left\{\frac{N_{infected,r,l}}{N_{infected,total,p}}\times \frac{1-\exp\left(-N_{virus,k,r,l}\times \alpha_{k}\right)}{\sum_{k,r}\left[1-\exp\left(-N_{virus,k,r,l}\times \alpha_{k}\right)\right]}\right\}\;,$$ [3]

where k represents four considered scenarios for infection transmission modes, including direct droplet deposition, fomite, long-range aerosol inhalation, and short-range aerosol inhalation; r, two considered microenvironments in the cruise ship including cabins and public areas; p, three considered simulation periods including during the entire outbreak duration, before the passenger quarantine began, and after the passenger quarantine began; $C_{infection,\;k,r,p}$, infection contribution associated with transmission mode k in microenvironment r in simulation period p; $D_{p}$, number of simulation days in the simulation period p (i.e., 36, 16, and 20 for the entire outbreak duration before all passengers disembarked, before the passenger quarantine began, and after the passenger quarantine began, respectively); $N_{infected,r,l}$, number of infected cases in microenvironment r on day l of the simulation period; $N_{infected,total,p}$, total number of infected cases in the cruise ship during the simulation period p; $N_{virus,k,r,l}$, number of SARS-CoV-2 RNA copies that reached the relevant respiratory tract region (i.e., LRT for inhalation and URT for direct deposition and fomite) via transmission mode k in microenvironment r on day l of the simulation period; and $\alpha_{k}$, infectivity of SARS-CoV-2 for the target respiratory tract (i.e., LRT for inhalation and URT for direct deposition and fomite).

This approach allows for summarizing estimates of infection contributions by transmission mode, contact range, microenvironment (i.e., public areas or passenger cabins), and/or simulation period independently, as needed.

Short-range transmission occurs by direct deposition of respiratory droplets and inhalation of aerosols only when susceptible individuals were within a defined close-range contact area of infected individuals. The close-range contact area was defined assuming a conical area in front of an infector with the head angle of 60° and length of 3 m (described in detail in *SI Appendix*, section 1.2.2) (42, 50). The projected surface area of the cone on the floor was ∼4.7 m^2^, which is equivalent to a surface area of a circle around the infector with a radius of ∼1.2 m. The probability that a susceptible individual was present within the close-contact cone was estimated based on the proportion of the zone surface area to the projected surface area of the cone on the floor (*SI Appendix*, section 1.2.2).

Long-range inhalation transmission occurs via inhalation of aerosols when susceptible individuals were outside the close-contact area. Fomite transmission occurs when susceptible individuals came in contact with contaminated surfaces, which could be contaminated by infected individuals through direct touching, direct deposition of respiratory droplets, and/or deposition of respiratory aerosols at any time point and location in the model framework.

### Combining the Transmission Risk Model with a Developed Epidemic Model.

The mechanistic infection transmission model was combined with a modified version of the Reed–Frost epidemic model to simulate the transmission of COVID-19 aboard the ship. We assumed that 1) the infection is spread from infected individuals to others by four main transmission pathways (long-range inhalation, short-range inhalation, direct deposition within close range, and fomite contact), 2) a portion of susceptible individuals in the group will develop the infection and will be infectious to others (the portion of “susceptibles” who will develop the infection is estimated by the transmission risk model), 3) the probability of coming into adequate contact with any other specified individual in the group within one time interval depends on the interaction behavior of the individual and is estimated using the Markov chain method, 4) the susceptible individuals in the cruise ship were isolated from others outside the cruise ship, and 5) these conditions remain constant during one whole day of the outbreak.

To estimate the spread of the disease, we estimated the number of infected cases among susceptible individuals, some of whom were cabinmates with infected individuals and some were not, at the end of each simulation day using the transmission risk model. The infected cases were assumed to develop infection and become “infectors” after the latent period, which was estimated by reducing the assumed effective subclinical infectious period (i.e., the time span between the onset of the infectious period and the appearance of clinical signs of disease) from the effective incubation period (i.e., the time span between infection and detection among infected cases). The number of cabins with at least one infected individual (i.e., “infected cabins”) was calculated at the end of each simulation day by assuming the number of newly infected cabins is equal to the number of newly infected cases who were not in one of the previously infected cabins at the beginning of the simulation day. The numbers of susceptible individuals who were not cabinmates with an infector $\left(N_{susceptibles-common}\right)$ and susceptible individuals inside the infected cabins $\left(N_{susceptibles-cabin}\right)$ at the beginning of each simulation day (d) were estimated using Eqs. 4 and **5** (except for the first period of infection transmission):

$$N_{susceptibles-common}\left(d\right)=N_{total-onboard}-\left[N_{infected-cabin}\left(d\right)\times N_{average-cabin}\right],$$ [4]

$$N_{susceptibles-cabin}\left(d\right)=\left[N_{infected-cabin}\left(d\right)\times N_{average-cabin}\right]-N_{infector}\left(d\right)-\sum_{i=0}^{d-1}N_{detected-cases}\left(i\right),$$ [5]

where $N_{total-onboard}$ represents total number of passengers and crew onboard (constant during the outbreak); $N_{infected-cabin}$, estimated number of infected cabins at the beginning of each day; $N_{average-cabin}$, average number of individuals in one cabin; $N_{infector}$, number of infectors; and $\sum N_{detected-cases}$, cumulative number of detected infected cases or disembarked individuals from the cruise ship.

We assumed the infected cases could spread infectious virus until only 1 d after the incubation period because the passengers were screened daily and removed from the cruise ship if they had shown symptoms or positive test results. We divided the transmission patterns into four periods, each of which having different epidemic characteristics, as described in *SI Appendix*, section 1.1. Several checkpoint conditions were introduced to the epidemic model to ensure reasonable bounds (*SI Appendix*, section 2.3).

### Analysis.

The model framework requires numerous assumptions or estimates for unknown or uncertain input parameters, which were culled from existing literature where possible and otherwise estimated or assumed using known information about the *Diamond Princess* cruise ship. Because there is high uncertainty around several critical model parameters, we utilized a scenario modeling approach in which values for unknown or uncertain epidemic and transmission modeling parameters were varied over a wide range of possibilities to generate a matrix of possible solutions. In this approach, our estimates of ID_50_ and infectivity for aerosol inhalation (assuming deposition in the LRT) and fomite and droplet deposition (assuming deposition in the URT) play a critical role in understanding the transmission of airborne infectious diseases. As we are not aware of any clinical studies to date that report these values for SARS-CoV-2 in humans or animals, we rely on our model approach to “back-calculate” effective ID_50_ for upper and lower respiratory tracts (on a basis of RNA copies) for each considered scenario from the first 5 d of the simulation period called the “calibration period.” A total of 21,600 scenarios were modeled across a range of estimates or assumptions for eight critical unknown or uncertain input parameters (Table 1). Estimates and assumptions for these parameters are described in detail in *SI Appendix*, section 3. We ran the model with each possible combination of the eight unknown or uncertain input parameters shown in Table 1 (10 × 5 × 6 × 3 × 3 × 2 × 2 × 2 = 21,600) in order to search a wide range of possible parameter values and combinations of parameter values.

**Table 1.** Summary of the ranges of eight unknown or uncertain critical model input parameters that defined each model iteration

Model inputs	Epidemiological factors			Mechanistic transmission factors				
	Effective incubation period	Effective subclinical infectious period	Effective reproduction no. for the index case	Symptomatic vs. asymptomatic emissions	Ratio of aerosol vs. droplet emissions	Minimum close interaction time in cabins	Quarantine infection control efficiency	URT/LRT ID_50_ ratio
No. scenarios	10	5	6	2	3	2	2	3
Range	6–15 (days)	1–5 (days)	1–6	0.544 1.0	0.3:1	8 or 12 h per day	Moderate High	1:1
					2.4:1			10:1
					1:1			100:1

## Results

**Fig. 1.**
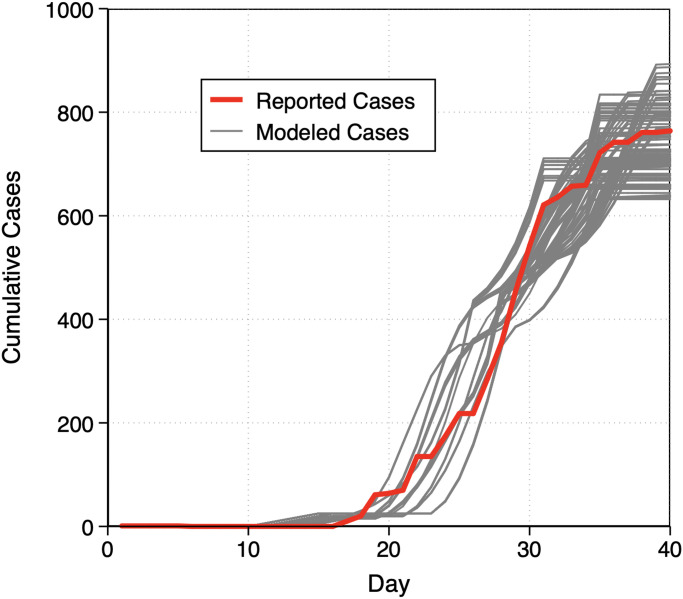
Reported (actual) and modeled (predicted) cumulative COVID-19 cases aboard the *Diamond Princess* cruise ship from January 20 to February 24, 2020. Modeled cases are from 132 model scenarios that met acceptable criteria (*R*^2^ > 0.95 for cumulative daily cases and *R*^2^ > 0 for daily cases).

A total of 132 model iterations met the acceptability criteria of *R*^2^ > 0.95 for daily cumulative cases and *R*^2^ > 0 for daily cases (0.6% of the total number of model iterations). The cumulative number of infected cases reported in various outlets was 765 cases; the average (±SD) cumulative number of modeled infected cases among iterations meeting acceptability criteria was 736 (±64) (Fig. 1). A total of 611, 495, and 323 model scenarios achieved *R*^2^ > 0 for daily cases and less stringent criteria of *R*^2^ > 0.8, 0.85, and 0.9 for daily cumulative cases, respectively.

Table 2 shows the number of acceptable iterations that were associated with a specific assumption for each of the eight unknown or uncertain model input parameters, as well as the average *R*^2^ value for those iterations. Table 2 also shows the mean numerical estimate of each of these model input parameters, which demonstrates a “best estimate” for each parameter using this approach.

**Table 2.** Distribution of acceptable model iterations (defined as *R*^2^ > 0.95 between reported and modeled daily cumulative case numbers and nonnegative *R*^2^ for daily case numbers) that were associated with a specific assumption for eight unknown or uncertain model input parameters. The boldface type in the table shows model input scenarios with the largest number of acceptable iterations

	Model input scenarios*					
Model inputs	No. of acceptable iterations (average *R*^2^)					Best estimates (mean ± SD)
Effective incubation period	10 d	11 d	**12 d**	13 d	14 d	11.9 ± 1.3
	25 (0.95)	30 (0.97)	**31 (0.98)**	31 (0.97)	15 (0.96)	
Effective subclinical infectious period	2 d	3 d	4 d	**5 d**		4.2 ± 1.1
	14 (0.95)	30 (0.97)	1 (0.98)	**87 (0.97)**		
Asymptomatic/symptomatic emission scenarios^†^	A/S = 0.545	**A/S = 1.0**				0.78 ± 0.23
	64 (0.96)	**68 (0.98)**				
Emission rate scenarios (aerosol/droplet ratio)	A/D = 0.3	**A/D = 2.4**	A/D = 1.0			A/D = 1.3 ± 0.9^‡^
	42 (0.97)	**50 (0.97)**	40 (0.97)			
Minimum close interaction time in the cabins	**8 h**	6 h				11.9 ± 4.0
	**68 (0.97)**	64 (0.97)				
Effective reproduction no. for the index case	R_Eff_ = 2	R_Eff_ = 3	**R**_**Eff**_ **= 4**	R_Eff_ = 5		3.9 ± 0.9
	11 (0.96)	30 (0.97)	**53 (0.97)**	38 (0.97)		
URT/LRT ID_50_ ratio scenarios	Ratio = 1	Ratio = 10	Ratio** = 100**			47.1 ± 46.9
	35 (0.97)	39 (0.97)	**58 (0.97)**			
Infection control efficiency scenarios	**Moderate**	High				Moderate^§^
	**70 (0.97)**	62 (0.97)				

* Scenarios with no acceptable model iterations were omitted from this table.

^†^ Asymptomatic refers to both asymptomatic and presymptomatic individuals.

^‡^ The proportion of SARS-CoV-2 RNA copies emitted in form of inhalable aerosols (A) to large droplets (D).

^§^ Nonnumerical; thus, no number could be attributed as the mean value.

Some estimates or assumptions for individual input parameters resulted in a larger proportion of successful model scenarios associated with that input compared to others (e.g., URT/LRT ID_50_ of 100:1, effective reproduction number of 4, effective subclinical infectious period of 5), which suggests that although these values may not be precise estimates or assumptions, they may be reasonably representative of the central tendencies of these parameters. Other parameters had similar numbers of successful model iterations associated with each assumed value, including effective incubation period, the ratio between asymptomatic (or presymptomatic) and symptomatic emission rates, aerosol/droplet emission ratios, minimum close interaction times in cabins, and infection control efficacy, which suggests that these parameters still have a high degree of uncertainty and/or may be less important for model sensitivity.

**Fig. 2.**
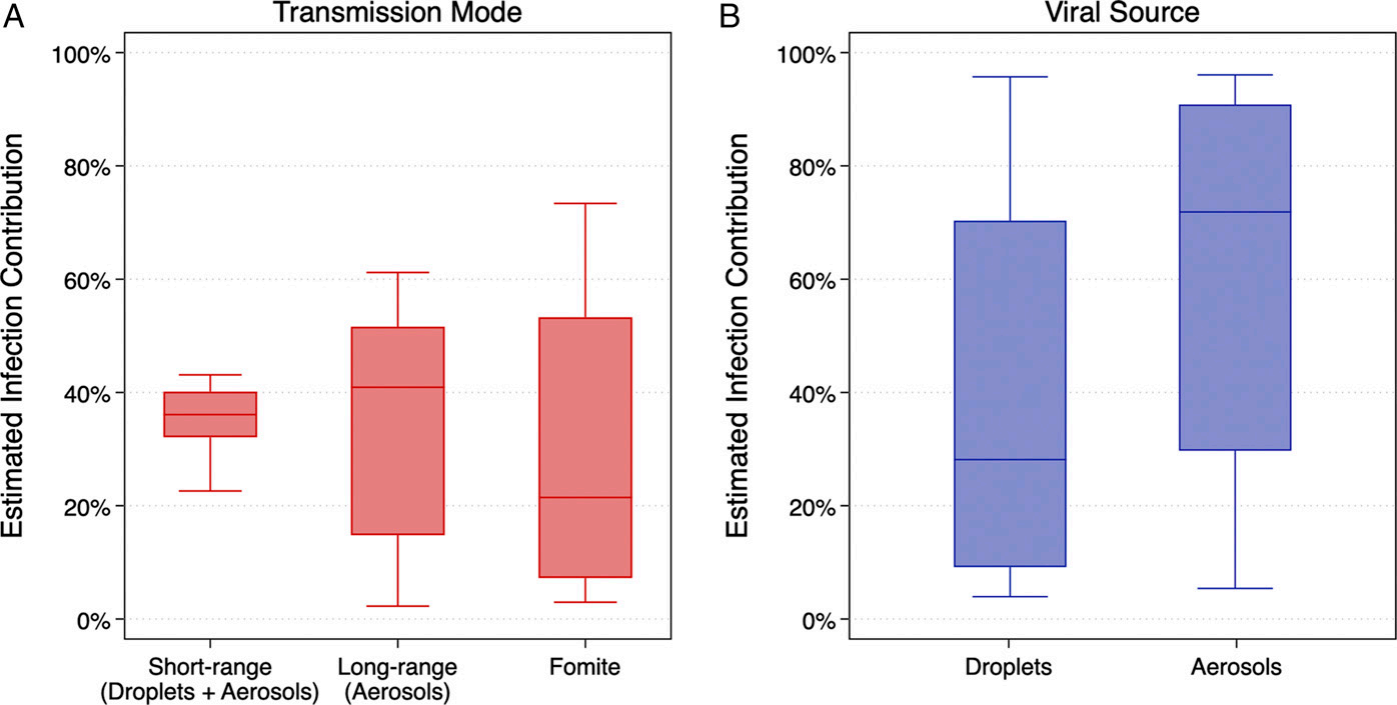
Estimates of the contributions of transmission modes (*A*) and viral sources (*B*) to infected cases aboard the *Diamond Princess* cruise ship over the entirety of the simulation period.

Fig. 2 shows distributions of the estimated contributions of each transmission mode and viral source to the progression of COVID-19 aboard the ship over the entire duration that passengers remained aboard. Among the model scenarios meeting acceptability criteria, median (mean) estimates of the contributions of short-range (i.e., droplets and aerosols within close range), long-range (i.e., aerosols outside of close-range contact), and fomite transmission modes to infected cases aboard the ship were 36% (35%), 41% (35%), and 21% (30%), respectively (Fig. 2*A*). The estimated contribution of short-range (droplet plus aerosol) transmission did not exceed 44% in any of the model scenarios that met acceptability criteria, while individual model scenarios exceeded 61% and 73% for long-range aerosol and fomite transmission, respectively. Conversely, the estimated contribution of short-range (droplet plus aerosol) transmission was never lower than 22% for a single model scenario, while the estimated contributions of both long-range aerosol and fomite transmission were as low as 3% each, suggesting that the model framework yields a lower uncertainty in the contribution of short-range transmission than both long-range and fomite transmission. However, the central tendency of the most successful model iterations suggests that long-range aerosol and short-range aerosol plus droplet transmission represented similar contributions to infection cases aboard the cruise ship, on average, while the contribution of fomites was likely smaller.

Median (mean) estimates of the contributions of larger droplets (which includes only short-range and fomite transmission in the model framework) and smaller aerosols (which includes all possible modes of transmission) were 28% (41%) and 72% (59%), respectively (Fig. 2*B*). Differences between droplet and aerosol transmission were significant (Mann–Whitney *U* test, *P* < 0.0001). Individual model scenarios resulted in at least one scenario in which only one viral source dominated the other (up to 96% for each mode), but the central tendencies again suggest that smaller respiratory aerosols contributed a greater proportion to infection transmission aboard the cruise ship, on average, across all time periods (i.e., both before and after passenger quarantine).

**Fig. 3.**
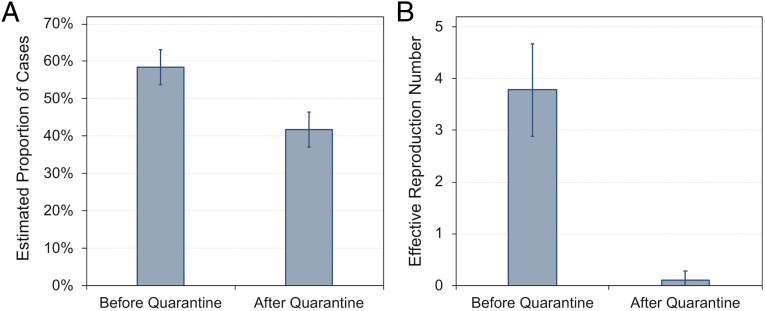
Mean (SD) estimates of (*A*) the proportion of cases and (*B*) the effective reproduction number before and after passenger quarantine.

Next, we analyzed the model results for periods before and after passenger quarantine started. Analyzing only the 132 model iterations that met acceptability criteria, the average (±SD) estimated proportion of cases that were transmitted prior to and after the passenger quarantine period was 58% (±5%) and 42% (±5%), respectively (Fig. 3*A*). The average (±SD) estimated effective reproduction number before and after the quarantine period was 3.8 (±0.9) and 0.1 (±0.2), respectively (Fig. 3*B*).

**Fig. 4.**
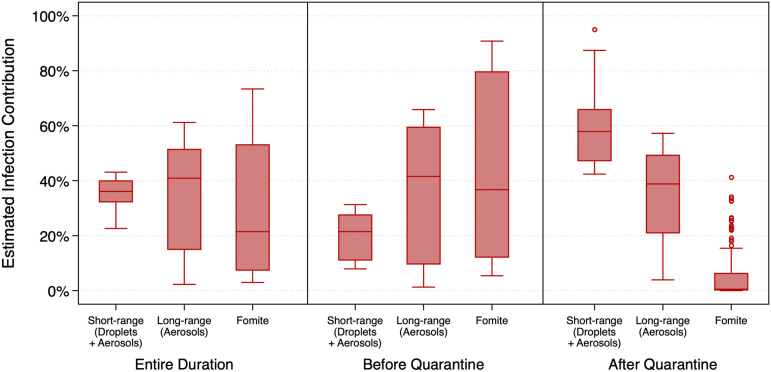
Estimates of the contribution of multiple transmission modes to infected cases aboard the *Diamond Princess* cruise ship over the entirety of the simulation period as well as before and after quarantine measures.

Estimates of the contributions of the specific transmission modes considered herein varied between the time periods before and after the passenger quarantine was in place (Fig. 4). Prior to the passenger quarantine period, when passengers were free to move about both cabin and public areas, median (mean) estimates of the contribution of long-range, fomite, and short-range transmission were 42% (34%), 37% (46%), and 22% (19%), respectively, suggesting that close-contact transmission contributed the least to overall transmission, while long-range aerosol and fomite transmission were likely similar in magnitude. Conversely, after the quarantine period began and passengers primarily remained in their cabins, the median (mean) estimates of the contribution of long-range, fomite, and short-range transmission were 39% (36%), 0.5% (6%), and 58% (59%), respectively, suggesting that close-contact transmission (via both droplets and aerosols) dominated during this time period, as expected. Before the quarantine, only the differences between short- and long-range transmission (Mann–Whitney *U* test, *P* < 0.0001) and between long-range and fomite transmission (Mann–Whitney *U* test, *P* = 0.0004) were significant. After the quarantine, all transmission mode comparisons were significant (*P* < 0.0001).

**Fig. 5.**
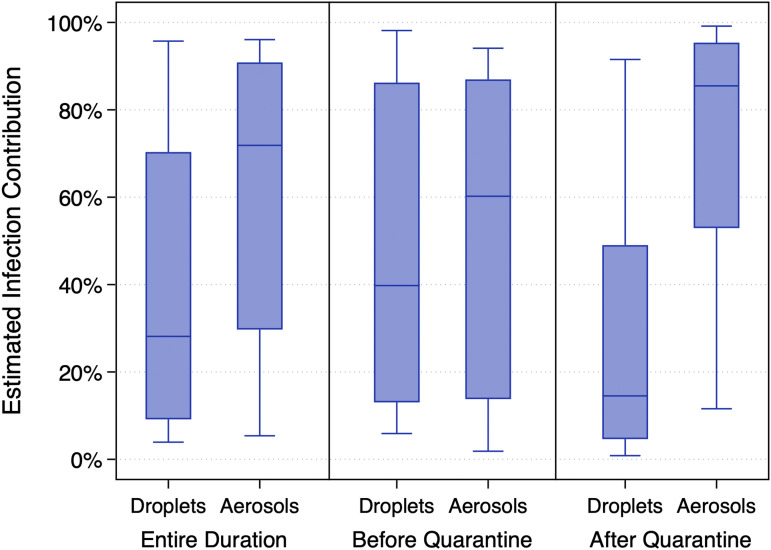
Estimates of the contribution of droplets and aerosols to infected cases aboard the *Diamond Princess* cruise ship over the entirety of the simulation period as well as before and after quarantine measures.

Estimates of the contributions of the different viral sources considered herein (i.e., droplets vs. aerosols) also varied between the time periods before and after the passenger quarantine was in place (Fig. 5). Median (mean) estimates of the contribution of droplets and aerosols prior to the passenger quarantine were 40% (50%) and 60% (50%) (*P* = 0.32), respectively, suggesting that both larger respiratory droplets and smaller respiratory aerosols contributed approximately equally to infected cases aboard the ship during this time period. Conversely, median (mean) estimates of the contribution of droplets and aerosols after the passenger quarantine began were 15% (27%) and 85% (73%) (*P* < 0.0001), respectively, suggesting that even though short-range transmission likely dominated during this period (Fig. 4), smaller aerosol transmission likely accounted for the vast majority of infected cases postquarantine, rather than larger droplets.

## Discussion

Our results show that airborne transmission by small aerosols containing SARS-CoV-2 was most likely the dominant mode of COVID-19 transmission aboard the ship, even with assumptions of a very high ventilation rate (9 to 12 air changes per hour) and no air recirculation, which are both conservative assumptions that favor against long-range airborne transmission. The long-range and short-range transmission routes had similar contributions to the total number of infected cases. However, aerosol transmission across both short- and long-range distances accounted for >50% of disease transmission overall, which is contrary to the prevailing positions on how COVID-19 is spread held by organizations like WHO and CDC, but is consistent with emerging evidence for airborne transmission. Although there is high uncertainty around numerous model parameters, the model approach is designed to identify the most likely values of several unknown or uncertain parameters by analyzing only those model results that met acceptability criteria, and thereby providing insight into the likely importance of the various modes of transmission included in the framework without needing to know many of the unknown parameters.

The estimated contribution of fomite transmission before the quarantine started in the cruise ship (Fig. 2) was higher than the fomite contribution estimated after the quarantine started, and is also higher than another recent Markov model applied to health care settings (51). We believe this is primarily because of the unique conditions that exist in cruise ship environments. For example, in the *Diamond Princess* cruise ship, we know that almost half of the public areas (∼17,000 m^2^ out of total ∼35,000 m^2^) were outdoors, where there was minimal risk of airborne transmission; therefore, the contribution of fomite transmission was estimated to be higher before quarantine compared to after quarantine, after which passengers and crew spent most of their time indoors. Moreover, after the quarantine started, we assumed that people washed their hands more frequently and effectively in compliance with recommendations at the time. Moreover, if we assumed that SARS-CoV-2 can transmit only through fomites, our best estimate of the number of infected cases in the cruise ship was only 98 cases out of 3,711 passengers and crew members (i.e., 2.6%; *SI Appendix*, Table S5), compared to actual cases of over 700. For comparison, our estimate of the fomite-only attack rate of SARS-CoV-2 was lower than previous estimates of the norovirus attack rate via fomites in cruise ships (52, 53).

Although cruise ships represent unique built environments with presumably high ventilation rates and filtration, these findings underscore the importance of implementing public health measures that target the control of inhalation of small aerosols in addition to ongoing measures targeting control of large droplet and fomite transmission. Moreover, our best estimates of the model parameters may be reasonably representative of the central tendencies of these parameters, particularly for estimates or assumptions of individual input parameters that resulted in a larger proportion of successful model scenarios associated with that input compared to others as shown in Table 2.

We also conducted sensitivity analyses on the model results, described in detail in *SI Appendix*, section 4. Briefly, our sensitivity analyses demonstrate that 1) aerosol transmission alone provides the strongest association between measured and reported cases in a mode elimination analysis (*SI Appendix*, section 4.2); 2) primary epidemiological inputs among acceptable iterations most commonly clustered around effective subclinical infection periods of 5 d (with some 2 to 3 d) and effective incubation periods of 11 to 13 d (*SI Appendix*, section 4.3); 3) the ratio between the median infectious dose associated with URT and LRT is a critical factor in the model and remains to be better understood from clinical investigations (*SI Appendix*, section 4.4); and 4) the ratio for aerosol-to-droplet emissions remains an uncertain parameter but has less influence on the results than the URT/LRT ID_50_ assumptions (*SI Appendix*, section 4.5).

There are several limitations to this modeling approach. For one, there is considerable uncertainty in our model inputs, as numerous estimates, assumptions, and implications were made because of a lack of available information, especially related to COVID-19 epidemic and mechanistic transmission characteristics, the interactions among individuals onboard the ship, and the effectiveness of infection control strategies adopted during the quarantine period. Some of these assumptions could have a significant impact on the results. For example, while the average contribution of fomite transmission among acceptable model iterations was estimated to be lower than other the other two pathways, under some specific assumptions (e.g., ID_50,URT_/ID_50,LRT_ = 1; *SI Appendix*, section 4.4) or transmission periods (e.g., before passenger quarantine started when people spent considerably more time outdoors), fomite transmission could have been the dominant transmission mode (i.e., long-range airborne transmission is unlikely outdoors). Second, the model approach assumes constant and/or average values for numerous inputs in a given model iteration (e.g., every passenger was assumed to have the same probabilities of close-range contact with others and every infected individual was assumed to have the same emission rates of droplets in aerosols). By not considering variability in these parameters, we cannot directly account for “superspreader” events and any underlying biological, physical, or behavioral differences in those individuals involved. Instead, the model framework produces average and uniform outcomes, which remains a limitation. Third, we relied on a conventional discrete size cutoff to define aerosols and droplets (i.e., ∼10 µm); however, respiratory droplets and aerosols actually exist on a continuum of particle sizes influenced by inertia, gravitational settling, and evaporation, and there is increasing recognition that the distinction between droplet and aerosol is a false dichotomy that is inconsistent with our understanding of the physics of respiratory aerosols (15). We recognize and understand this as well, although we still find value in defining our model framework around this conventional definition because it aligns with the definitions in current public health guidance. It is worth noting that had we defined droplets as true “ballistic” droplets >100 µm, the estimated contribution of droplets to transmission would likely be substantially lower.

The model could also not consider the direct impacts of potentially influential characteristics such as sunlight, varying environmental conditions, or not-well-mixed conditions in the control volumes considered herein. We also did not define model scenarios based on some key physical or biological factors such as the impact of temperature, humidity, or exhaled air turbulence, many of which remain unknown or challenging to quantify in the framework, but many of which were unlikely to vary during the simulation period. However, the impacts of these parameters on the total number of cases were indirectly considered by calibration of the model results with the reported effective reproduction numbers when the index case was onboard the cruise ship (i.e., during the calibration period between January 20 and 25, 2020). Moreover, to estimate the potential effects of some of these indirectly considered parameters and processes on the contribution of various infection transmission pathways, we performed a sensitivity analysis on how much the model results would change if the combined impacts of them eliminate the risk of infection through one or two transmission pathways (*SI Appendix*, section 4.2). As more information becomes available, the model framework should continue to be tested and applied to other built environment transmission case studies.

Finally, the model framework developed in this study has utility for cruise ships, other indoor environments, and other infectious diseases. Specific to cruise ships, our results suggest that rapid masking, rapid identification of cases, and better isolation and quarantine practices could reduce the number of total cases significantly. With regard to relevance beyond cruise ships, the model can be applied to other indoor environments (e.g., hospitals, offices, and schools) to estimate the relative risk reduction potential for various intervention strategies. Absolute risk in these environments can also be modeled, along with the effectiveness of infection risk mitigation strategies, with the caveat that the underlying model is based on an outbreak scenario and unique combination of infectivity and susceptible adults. Last, this model approach has broad applicability beyond COVID-19 and cruise ships and can be used for estimating the contribution of transmission pathways of other airborne infectious diseases such as measles, tuberculosis, and influenza in other infection outbreaks.

## Supplementary Material

Supplementary File

## Data Availability

Code have been deposited in Zenodo (https://zenodo.org/record/3955528#.XxfUqp5KjIU).

## References

[1] D. Lewis, Is the coronavirus airborne? Experts can’t agree. Nature 580, 175 (2020). 32242113 10.1038/d41586-020-00974-w

[2] WHO, Modes of transmission of virus causing COVID-19: Implications for IPC precaution recommendations. Scientific Brief (2020). https://www.who.int/news-room/commentaries/detail/modes-of-transmission-of-virus-causing-covid-19-implications-for-ipc-precaution-recommendations. Accessed 22 July 2020.

[3] CDC, How COVID-19 spreads (2020). https://www.cdc.gov/coronavirus/2019-ncov/prevent-getting-sick/how-covid-spreads.html. Accessed 25 November 2020.

[4] CDC, Scientific brief: SARS-CoV-2 and potential airborne transmission. Coronavirus disease 2019 (COVID-19) (2020). https://www.cdc.gov/coronavirus/2019-ncov/more/scientific-brief-sars-cov-2.html. Accessed 25 November 2020.

[5] L. Morawska, J. Cao, Airborne transmission of SARS-CoV-2: The world should face the reality. Environ. Int. 139, 105730 (2020). 32294574 10.1016/j.envint.2020.105730 PMC7151430

[6] P. Bahl., Airborne or droplet precautions for health workers treating COVID-19? J.Infect. Dis., 10.1093/infdis/jiaa189 (2020). PMC7184471 32301491

[7] K. A. Prather, C. C. Wang, R. T. Schooley, Reducing transmission of SARS-CoV-2. Science 368, 1422–1424 (2020). 32461212 10.1126/science.abc6197

[8] L. Morawska., How can airborne transmission of COVID-19 indoors be minimised? Environ. Int. 142, 105832 (2020). 32521345 10.1016/j.envint.2020.105832 PMC7250761

[9] S. J. Dancer., Putting a balance on the aerosolization debate around SARS-CoV-2. J. Hosp. Infect. 105, 569–570 (2020). 32405126 10.1016/j.jhin.2020.05.014 PMC7219351

[10] S. Asadi, N. Bouvier, A. S. Wexler, W. D. Ristenpart, The coronavirus pandemic and aerosols: Does COVID-19 transmit via expiratory particles? Aerosol Sci. Technol. 0, 1–4 (2020). 32308568 10.1080/02786826.2020.1749229 PMC7157964

[11] National Academies of Sciences, Engineering, and Medicine, Rapid Expert Consultation on SARS-CoV-2 Viral Shedding and Antibody Response for the COVID-19 Pandemic (April 8, 2020) (National Academies Press, 2020).

[12] E. A. Nardell, R. R. Nathavitharana, Airborne spread of SARS-CoV-2 and a potential role for air disinfection. JAMA 324, 141–142 (2020). 32478797 10.1001/jama.2020.7603

[13] J. Allen, L. Marr, Re-thinking the potential for airborne transmission of SARS-CoV-2. 10.20944/preprints202005.0126.v1 (15 June 2020).

[14] L. Morawska, D. K. Milton, It is time to address airborne transmission of Coronavirus disease 2019 (COVID-19). Clinical Infect. Dis. 71, 2311–2313 (2020). 32628269 10.1093/cid/ciaa939 PMC7454469

[15] F. C. Fang., COVID-19—lessons learned and questions remaining. Clinical Infect. Dis., 10.1093/cid/ciaa1654 (2020). PMC7797746 33104186

[16] ASHRAE, “ASHRAE issues statements on relationship between COVID-19 and HVAC in buildings” (2020). https://www.ashrae.org/about/news/2020/ashrae-issues-statements-on-relationship-between-covid-19-and-hvac-in-buildings. Accessed 22 July 2020.

[17] L. Bourouiba, Turbulent gas clouds and respiratory pathogen emissions: Potential implications for reducing transmission of COVID-19. JAMA 323, 1837–1838 (2020). 32215590 10.1001/jama.2020.4756

[18] V. Stadnytskyi, C. E. Bax, A. Bax, P. Anfinrud, The airborne lifetime of small speech droplets and their potential importance in SARS-CoV-2 transmission. Proc. Natl. Acad. Sci. U.S.A. 117, 11875–11877 (2020). 32404416 10.1073/pnas.2006874117 PMC7275719

[19] R. Tellier, Y. Li, B. J. Cowling, J. W. Tang, Recognition of aerosol transmission of infectious agents: A commentary. BMC Infect. Dis. 19, 101 (2019). 30704406 10.1186/s12879-019-3707-y PMC6357359

[20] P. Y. Chia.; Singapore 2019 Novel Coronavirus Outbreak Research Team, Detection of air and surface contamination by SARS-CoV-2 in hospital rooms of infected patients. Nat. Commun. 11, 2800 (2020). 32472043 10.1038/s41467-020-16670-2 PMC7260225

[21] Y. Liu., Aerodynamic analysis of SARS-CoV-2 in two Wuhan hospitals. Nature 582, 557–560 (2020). 32340022 10.1038/s41586-020-2271-3

[22] J. L. Santarpia., Aerosol and surface contamination of SARS-CoV-2 observed in quarantine and isolation care. Sci. Rep. 10, 12732 (2020). 32728118 10.1038/s41598-020-69286-3 PMC7391640

[23] J. A. Lednicky., Collection of SARS-CoV-2 virus from the air of a clinic within a university student health care center and analyses of the viral genomic sequence. Aerosol Air Qual. Res. 20, 1167–1171 (2020). 33424954 10.4209/aaqr.2020.02.0202 PMC7792982

[24] J. A. Lednicky., Viable SARS-CoV-2 in the air of a hospital room with COVID-19 patients. Int. J. Infect. Dis. 100, 476–482 (2020). 32949774 10.1016/j.ijid.2020.09.025 PMC7493737

[25] K. Nissen., Long-distance airborne dispersal of SARS-CoV-2 in COVID-19 wards. Sci. Rep. 10, 19589 (2020). 33177563 10.1038/s41598-020-76442-2 PMC7659316

[26] Y. Li., Evidence for probable aerosol transmission of SARS-CoV-2 in a poorly ventilated restaurant. Infectious Diseases (except HIV/AIDS). 10.1101/2020.04.16.20067728 (31 May 2020).

[27] S. L. Miller., Transmission of SARS‐CoV‐2 by inhalation of respiratory aerosol in the Skagit Valley Chorale superspreading event. Indoor Air, 10.1111/ina.12751 (2020). PMC7537089 32979298

[28] Y. Shen., Community outbreak investigation of SARS-CoV-2 transmission among bus riders in Eastern China. JAMA Intern Med, 10.1001/jamainternmed.2020.5225 (2020). PMC7489377 32870239

[29] E. C. Dick, L. C. Jennings, K. A. Mink, C. D. Wartgow, S. L. Inhorn, Aerosol transmission of rhinovirus colds. J. Infect. Dis. 156, 442–448 (1987). 3039011 10.1093/infdis/156.3.442

[30] H. Lei., Routes of transmission of influenza A H1N1, SARS CoV, and norovirus in air cabin: Comparative analyses. Indoor Air 28, 394–403 (2018). 29244221 10.1111/ina.12445 PMC7165818

[31] A. N. M. Kraay., Fomite-mediated transmission as a sufficient pathway: A comparative analysis across three viral pathogens. BMC Infect. Dis. 18, 540 (2018). 30373527 10.1186/s12879-018-3425-x PMC6206643

[32] S. Xiao, Y. Li, T. W. Wong, D. S. C. Hui, Role of fomites in SARS transmission during the largest hospital outbreak in Hong Kong. PLoS One 12, e0181558 (2017). 28727803 10.1371/journal.pone.0181558 PMC5519164

[33] R. M. Jones, E. Adida, Influenza infection risk and predominate exposure route: Uncertainty analysis. Risk Anal. 31, 1622–1631 (2011). 21418085 10.1111/j.1539-6924.2011.01600.x

[34] B. Stephens., Microbial exchange via fomites and implications for human health. Curr. Pollution Rep. 5, 198–213 (2019). 10.1007/s40726-019-00123-6 PMC7149182 34171005

[35] L. F. Moriarty, Public health responses to COVID-19 outbreaks on cruise ships—worldwide, February–March. MMWR Morb. Mortal. Wkly. Rep. 69, 347–352 (2020). 32214086 10.15585/mmwr.mm6912e3 PMC7725517

[36] Worldometer, February 2020 coronavirus news updates—Worldometer (2020). https://www.worldometers.info/coronavirus/feb-2020-news-updates-covid19/. Accessed 3 April 2020.

[37] Princess Cruise Lines, Ltd., *Diamond Princess* updates—notices and advisories (2020). https://www.princess.com/news/notices_and_advisories/notices/diamond-princess-update.html. Accessed 2 April 2020.

[38] G. N. Sze To, C. Y. H. Chao, Review and comparison between the Wells-Riley and dose-response approaches to risk assessment of infectious respiratory diseases. Indoor Air 20, 2–16 (2010). 19874402 10.1111/j.1600-0668.2009.00621.x PMC7202094

[39] M. Wang, C.-H. Lin, Q. Chen, Advanced turbulence models for predicting particle transport in enclosed environments. Build. Environ. 47, 40–49 (2012).

[40] C. Chen, W. Liu, C.-H. Lin, Q. Chen, Comparing the Markov chain model with the Eulerian and Lagrangian models for indoor transient particle transport simulations. Aerosol Sci. Technol. 49, 857–871 (2015).

[41] M. Nicas, R. M. Jones, Relative contributions of four exposure pathways to influenza infection risk. Risk Anal. 29, 1292–1303 (2009). 19558389 10.1111/j.1539-6924.2009.01253.x

[42] M. Nicas, G. Sun, An integrated model of infection risk in a health-care environment. Risk Anal. 26, 1085–1096 (2006). 16948699 10.1111/j.1539-6924.2006.00802.x

[43] R. M. Jones., Characterizing the risk of infection from *Mycobacterium tuberculosis* in commercial passenger aircraft using quantitative microbial risk assessment. Risk Anal. 29, 355–365 (2009). 19076326 10.1111/j.1539-6924.2008.01161.x

[44] R. M. Jones, M. Nicas, Benchmarking of a Markov multizone model of contaminant transport. Ann. Occup. Hyg. 58, 1018–1031 (2014). 25143517 10.1093/annhyg/meu055

[45] R. M. Jones, M. Nicas, Experimental evaluation of a Markov multizone model of particulate contaminant transport. Ann. Occup. Hyg. 58, 1032–1045 (2014). 25135075 10.1093/annhyg/meu056

[46] M. Nicas, D. Best, A study quantifying the hand-to-face contact rate and its potential application to predicting respiratory tract infection. J. Occup. Environ. Hyg. 5, 347–352 (2008). 18357546 10.1080/15459620802003896 PMC7196690

[47] US Department of Homeland Security Science and Technology, Master question list for COVID-19 (caused by SARS-CoV-2): Weekly report (Hazard Awareness and Characterization Technology Center, 2020). https://www.dhs.gov/publication/st-master-question-list-covid-19. Accessed 22 July 2020.

[48] A. L. Hartman., SARS-CoV-2 infection of African green monkeys results in mild respiratory disease discernible by PET/CT imaging and shedding of infectious virus from both respiratory and gastrointestinal tracts. PLoS Pathog. 16, e1008903 (2020). 32946524 10.1371/journal.ppat.1008903 PMC7535860

[49] S. F. Sia., Pathogenesis and transmission of SARS-CoV-2 in golden hamsters. Nature 583, 834–838 (2020). 32408338 10.1038/s41586-020-2342-5 PMC7394720

[50] W. Chen, N. Zhang, J. Wei, H.-L. Yen, Y. Li, Short-range airborne route dominates exposure of respiratory infection during close contact. medRxiv:2020.03.16.20037291 (20 March 2020).

[51] R. M. Jones, Relative contributions of transmission routes for COVID-19 among healthcare personnel providing patient care. J. Occup. Environ. Hyg. 17, 408–415 (2020). 32643585 10.1080/15459624.2020.1784427

[52] E. T. Isakbaeva., Norovirus transmission on cruise ship. Emerg. Infect. Dis. 11, 154–158 (2005). 15705344 10.3201/eid1101.040434 PMC3294347

[53] R. Vivancos., Norovirus outbreak in a cruise ship sailing around the British isles: Investigation and multi-agency management of an international outbreak. J. Infect. 60, 478–485 (2010). 20359496 10.1016/j.jinf.2010.03.018

