# Supplementary material for: Mechanistic transmission modeling of COVID-19 on the Diamond Princess cruise ship demonstrates the importance of aerosol transmission
Source: Proc Natl Acad Sci U S A. 2021 Feb 3;118(8):e2015482118. doi: 10.1073/pnas.2015482118 (PMC7923347; doi:10.1073/pnas.2015482118)
Supplement: Supplementary File [file pnas.2015482118.sapp.pdf]

PNAS
www.pnas.org

Supplementary Information for

Mechanistic Transmission Modeling of COVID-19 on the Diamond Princess Cruise Ship Demonstrates the Importance of Aerosol Transmission

Parham Azimi,[1] Zahra Keshavarz,[1] Jose Guillermo Cedeno Laurent,[1] Brent R. Stephens,[2] Joseph G. Allen[1]

[1]Environmental Health Department, Harvard T.H. Chan School of Public Health, Boston, MA USA

[2]Department of Civil, Architectural, and Environmental Engineering, Illinois Institute of Technology, Chicago, IL USA.

* Parham Azimi; **Email:** pazimi@hsph.harvard.edu

* Joseph G. Allen; **Email:** jgallen@hsph.harvard.edu

This PDF file includes:

Supplementary text

Figures S1 to S8

Tables S1 to S8

SI References

Other supplementary materials for this manuscript include the following:

Not Applicable.

Table of Contents

1. Mechanistic Infection Transmission and Dose-Response Model Framework 3
1.1 Markov chain framework 3
1.2 Injection rates of SARS-CoV-2 to various states aboard the cruise ship......................10
1.3 Dose Response Model 13
1.4 Contribution from each transmission mode 14
2. Combining the Complex Infection Transmission Risk Model with a Developed Epidemic Model 15
2.1 Developing an adjusted Reed-Frost model for the cruise ship outbreak 15
2.2 Epidemic characteristics of the outbreak during four assumed infection transmission periods 17
2.3 Adding checkpoint conditions to the epidemic model 17
3. Identifying the primary unknown or uncertain epidemic and infection transmission modeling parameters 18
3.1 Effective incubation period 18
3.2 Effective subclinical infectious period for infectors in the cruise ship 19
3.3 Emission rate of SARS-CoV-2 from symptomatic and asymptomatic (or pre-symptomatic) cases 19
3.4 Emission rate of SARS-CoV-2 RNA copies in the form of large droplets and inhalable aerosols 20
3.5 Minimum close-range interaction time in the cabins 24
3.6 COVID-19 effective reproduction number for the index case.......................................24
3.7 SARS-CoV-2 infectious dose 24
3.8 Efficacy of infection control strategies during the quarantine period 25
4. Analyzing model outcomes and conducting sensitivity analyses.......................................26
4.1 Selecting acceptable model iterations 26
4.2 Model sensitivity to eliminating transmission modes using 'best estimates' of model parameters..28
4.3 Model sensitivity to primary epidemiological inputs 29
4.4 Model sensitivity to changes in the ratio between infectious dose for URT and LRT 30
4.5 Model sensitivity to changes in emission rate scenarios..32
4.6 Statistical significance testing on the model results..34
References 36

A combination of mechanistic infection transmission, dose-response model and epidemic models was adopted to estimate the contributions of specific infection transmission modes to the number of COVID-19 cases among individuals aboard the Diamond Princess Cruise Ship. Four main transmission pathways, including long-range inhalation, short-range inhalation, direct deposition, and fomite contact, were considered as the infection transmission mechanisms from infected individuals to others under a wide range of possible scenarios. Daily cumulative case counts from passengers aboard the ship are used as the primary model outcome. The model approach is designed to identify the most likely values of several unknown or uncertain parameters by analyzing only those model results that yield acceptable coefficients of determination (R^2) between reported and modeled daily cumulative and daily case numbers, and thereby providing insight into the likely importance of the various modes of transmission included in the framework.

Here we describe in detail: (1) the mechanistic infection transmission and dose-response model, (2) the epidemic model, (3) processes for identifying key unknown or uncertain model parameters, and (4) processes for analyzing model outcomes and conducting sensitivity analyses.

1. Mechanistic Infection Transmission and Dose-Response Model Framework

The mechanistic transmission model uses a Markov chain process to estimate the number of SARS-CoV-2 copies present in numerous physical states, as well as the probability of transmission of SARS-CoV-2 viruses between each defined state, aboard the ship over time. The transmission model is coupled with a dose-response model to predict the probability of infection to susceptible individuals onboard the ship over time.

1.1 Markov chain framework

A Markov chain is a random process that undergoes transitions from one state to another in a state space. Physical elements (e.g., room air and surfaces, human skin and mucus membranes, etc.) and pathogen removal mechanisms (e.g., loss of viability, ventilation, and filtration) in the source environment-receptor pathways are represented as "*states*" in a discrete-time Markov chain model. Pathogens can be transferred and exchanged between states due to physical mechanisms such as emission, deposition, resuspension, filtration, and ventilation. Markov chain models have been used previously for estimating doses of influenza virus in several environments including healthcare facilities and airplanes.(1–6)

Markov chain process consist of a Markov chain matrix (MCM), a distribution array showing the number of pathogens in considered "*states*" after a certain time, and an injection array showing the new number of pathogens injected to the system in each time step. To generate a MCM for a state space with n "*states*", first, we need to generate an $n \times n$ transmission rate matrix demonstrating the transmission or removal rates of pathogens between two considered "*states*" of i and j (λ_{ij}) in the state space. In the transmission rate matrix, the λ_{ii} values are considered equal to zero, as demonstrated in Figure S1. The overall rate at which a pathogen can leave state i (λ_i) is the sum of the rate constants for removal from that state.

$$\lambda_i = \sum_{j=1}^{n} \lambda_{ij} \qquad \textbf{Equation S1}$$

MCM is an $n \times n$ probability matrix demonstrating if a pathogen is in state i in time t, what the probabilities of remaining in the same state (P_{ii}) or moving to state j (P_{ij}) are in time $t + \Delta t$. P_{ii} and P_{ij} values can be estimated from Equation S2, and S3, respectively.

$$P_{ii} = e^{(-\lambda_i \times \Delta t)}$$

Equation S2

$$P_{ij} = \frac{\lambda_{ij}}{\lambda_i} \times (1 - P_{ii})$$

Equation S3

We considered time steps (Δt) of one second for the Markov chain process. If there are m time steps between two pathogen injections into the state space, the number of pathogens in each state after k injections can be estimated from the Equation S4.

$$D_k = \left[D_{k-1} \times MCM^{(m)}\right] + I_k$$

Equation S4

Where

D_k: Distribution array showing the number of pathogens in each state after k injections

I_k: Injection array showing the number of pathogens injected to each state at k^{th} injections

In this model, the Markov chain process was repeated for a one-day period and then a new MCM was generated for the next day until the end of the simulation period. We used PyCharm 2019.1.1 (Copyright © 2010-2020 JetBrains) with Python interpreter to deploy the process.

We considered 12 *states* for the Markov chain process as demonstrated in Figure S1. We considered two types of susceptible individuals aboard the ship: (i) uninfected individuals who were cabinmates of, and thus spent a significant amount of their time with, infected individuals (particularly after the passenger quarantine started), and (ii) uninfected individuals who were not cabinmates of infected individuals before they became infected. The list of states in Figure S1 excludes the air and surfaces of cabins with only uninfected individuals in them because no infectious virus is assumed to be present in cabins with uninfected individuals in them and defining these two additional states of indoor air and surfaces of these cabins would not change our calculations. The model then adjusts the number of cabins with infected individuals present at the end of each simulation day based on the number of new infected cases stemming from interactions in the common areas.

As reported, the COVID-19 outbreak was traced to a single passenger from Hong Kong who boarded the cruise ship in Yokohama on January 20 and then disembarked in Hong Kong on January 25. The first 10 cases were confirmed on February 4 after the ship arrived in the Yokohama port. The laboratory-confirmed cases of COVID-19 led to the quarantine of the Diamond Princess for 14 days beginning on February 5 at 7 am, with most passengers required to remain in their cabins. During February 16–23, nearly 1,000 persons were repatriated by air to their home countries, including 329 persons who returned to the United States and entered quarantine or isolation. During February 24 – March 1, the remaining crewmembers disembarked from the cruise ship.

Using the well-documented case information aboard the Diamond Princess Cruise Ship,(7) the transmission patterns of SARS-CoV-2 aboard the ship are divided into four distinct time periods:

1. January 20-25, 2020: when there was only one index case aboard the ship

2. January 25-February 5, 2020: the time between when the index case disembarked and before the passenger quarantine began
3. February 5-24, 2020: the time between the beginning of the passenger quarantine and the time when all passengers disembarked
4. February 24-March 1, 2020: the time between the disembarking of all passengers and the time when the remaining crew members disembarked

We applied the model only to the first three periods (January 20 through February 24, 2020) because detailed information was available on the daily counts of the number of people onboard and their infection status. We generated a new MCM for each day in this period to model mechanistic transmission and infection risk based on a number of assumptions for built environment parameters, crew and passengers' interactions, adopted infection control strategies, and the number of infectors and susceptible individuals estimated from application of the transmission risk model to the previous days. Figure S1 shows the transmission rate matrix that we used to generate the MCM for the first period of the outbreak as an example of the transmission rate matrices used in this modeling work. The gray cells demonstrate the possible transmission routes for SARS-CoV-2 between two *states* and the cell values are the transmission rates in units of inverse time.

The majority of epidemiological characteristics of the COVID-19 outbreak and built environment factors of the Diamond Princess Cruise Ship was culled from peer-reviewed journal articles, information provided by the cruise ship owner's website,(8) and CDC notifications and reports.(9, 10) However, some of the required parameters for the mechanistic infection transmission model, such as the rates of interactions among passengers and crew before the quarantine began and the built environment factors that were not reported in existing resources (e.g., the ventilation rate of cabins and public areas, inter-zonal airflow between cabins and public areas, etc.) were assumed to be similar to a typical cruise ship environment. Because we ultimately calibrate the model with the range of estimated effective reproduction numbers during the first distinct period, the impacts of our assumptions for unknown modeling parameters are limited.

Transmission/Removal Rates Per Hour		Cabin Air - Infected Cabins	Common Area Air	Cabin Surfaces - Infected Cabins	Common Area Surfaces	Palm Skin Cabinmate	URT-Cabinmate	LRT-Cabinmate	Palm Skin other people	URT-Other People	LRT-Other People	HVAC System	Inactivation - Removal
		1	2	3	4	5	6	7	8	9	10	11	12
Cabin Air - Infected Cabins	1	0	0.5	0.6	0	0	0	0.0	0	0	0.0	9.0	0.7
Common Area Air	2	0	0	0	0.6	0	0	0.0	0	0	0.0	12.0	0.7
Cabin Surfaces - Infected Cabins	3	0	0	0	0	0.0	0	0	0.0	0	0	0	0.1
Common Area Surfaces	4	0	0	0	0	0.0	0	0	0.0	0	0	0	0.1
Palm Skin Cabin-Mate	5	0	0	0.6	8.7	0	3.6	0	0	0	0	0	0.8
Upper Respiratory Tracts of Cabinmate	6	0	0	0	0	0	0	0	0	0	0	0	0
Lower Respiratory Tracts of Cabinmate	7	0	0	0	0	0	0	0	0	0	0	0	0
Palm Skin Other People	8	0	0	0.0	8.7	0	0	0	0	3.6	0	0	0.8
Upper respiratory Tracts of Other People	9	0	0	0	0	0	0	0	0	0	0	0	0
Lower respiratory Tracts of Other People	10	0	0	0	0	0	0	0	0	0	0	0	0
HVAC System	11	0.0	0.0	0	0	0	0	0	0	0	0	0	0.0
Inactivation - Removal	12	0	0	0	0	0	0	0	0	0	0	0	0

Figure S1. Example transmission rate matrix used for simulating the transmission of SARS-CoV-2 aboard the Diamond Princess Cruise Ship during the presence of the index case. Values are in units of inverse time.

Most of our assumed model parameters related to the characteristics of a typical cruise ship were culled from two prior studies. The first study we relied upon is Zheng et al., which modeled the risk of influenza on a typical cruise ship with 2000 passengers and 800 crew members using a Wells-Riley model.(11) Their model was validated using data from a previous influenza outbreak aboard a cruise ship from New York City in 1997 and was able to simulate the spread of the infection.(11) The second study we relied upon is Zhang et al., which modeled the transmission of infectious diseases via fomite contact in a typical cruise ship by considering seven functional areas including bench seats, restroom, retail counter, stair rails, dining table, dining chair, and handrail of viewing deck, as well as several types of contact surfaces.(12)

The following sub-sections describe several processes and assumptions for parameters used in the Markov chain model that relate to virus transmission and/or removal, primarily culled from a variety of literature sources relevant to cruise ship environments, human activity patterns, and virus viability.

1.1.1 Volume of surface area of various zones on the Diamond Princess Cruise Ship

We categorized the passenger-accessible areas of the cruise as either (i) cabins or (ii) common (public) areas such as restaurants, hallways, bars, galleries, gyms, casinos, and sport courts. The areas and volumes of public areas and cabins were estimated based on the information provided on the Princess Plus website.(8) We estimated the average floor area of the cabins to be ~22 m^2

using floor plans from the website. We estimated the floor area of indoor public areas to be ~18,000 m^2, located on Decks 5, 6, and 7. The floor area of hallways between cabins and outdoor zones accessible to passengers were estimated to be ~3,700 m^2 and ~17,000 m^2, respectively. The Princess Plus website did not provide the floor plan of other parts of the cruise ship where passengers did not have access, such as kitchens, control rooms, and the crew members' sleeping cabins. However, we assumed the floor area of those zones (except the sleeping cabins of crew members) was ~8000 m^2 (equal to the floor area of one deck). We also assumed that the indoor public areas were completely closed during the quarantine period, and that only half of the other indoor spaces were operating, with a total floor area of ~4000 m^2. To estimate space volumes, we assumed a ceiling height of 2.5 meters for cabins and hallways and 4 meters for other indoor public areas.

As the indoor public areas in the cruise ship were connected via hallways and stairways and the HVAC system was mixing the air in those spaces, we assumed the public area indoor spaces as a well-mixed single compartment. Although this assumption has limitations, in the absence of more detailed information on airflow pathways and interactions among individuals on the cruise ship, we found it a reasonable simplification. On the other hand, while each stateroom air was assumed well-mixed, we assumed different average exposure concentrations for non-infected and infected staterooms during one simulation day. The number of infected cabins was changed at the end of each simulation day based on the number of new infected cases in the public areas, as described in more detail in Section 2.1.

The floor plan of the public-accessible spaces in the Diamond Princess Cruise Ship including 14 decks out of 18 decks of the cruise ship was downloaded from the Princess Plus Company webpage and demonstrated in Figure S2.(8)

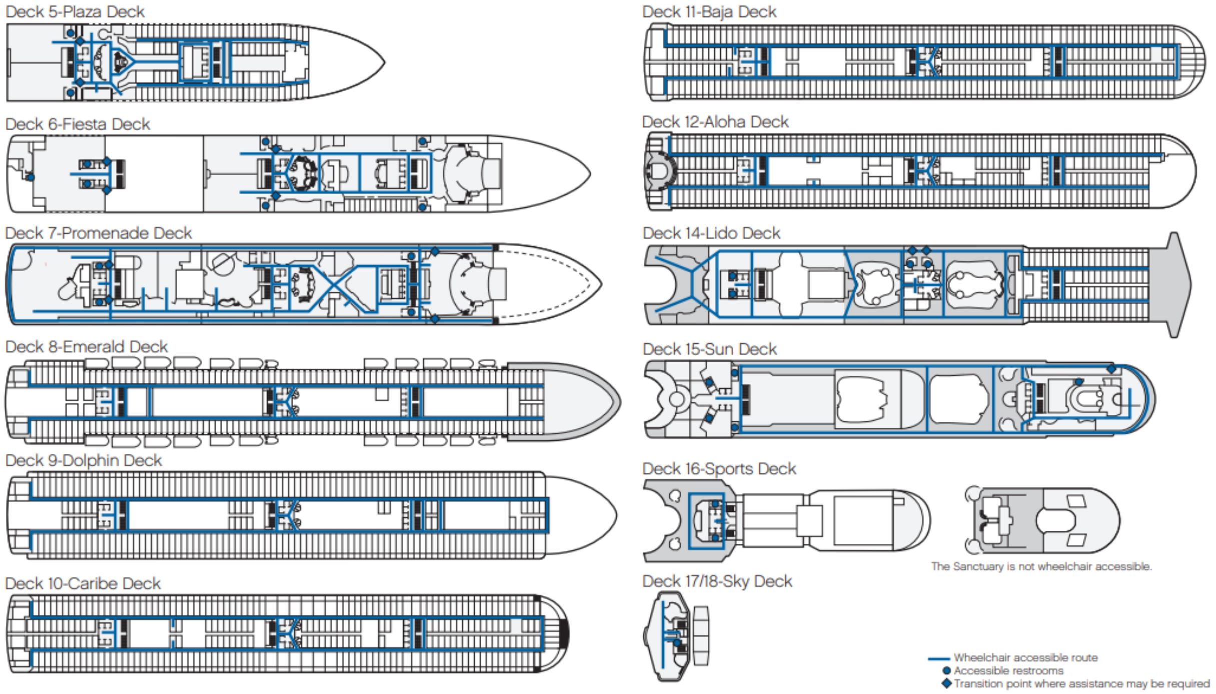

Figure S2. Floor plan of public-accessible decks of the Diamond Princess Cruise Ship

1.1.2 Ventilation and inter-zonal airflow rates in cabins and public areas

Our assumptions for the ventilation rate in the cruise ship were similar to Zheng et al., in which the air change rate was assumed to be 9 per hour in passenger and crew cabins and 12 per hour in other indoor locations (11). We also made a conservative assumption against the contribution of long-range transmission of infectious aerosols in that there was no air re-circulation in the cruise ship spaces and the supply air to the cabins and public area were 100% outdoor air (13). We assumed the cabins in the Diamond Princess Cruise Ship were positively pressurized and the cabin air were forced outside into hallways, which were considered as part of the public areas, similar to a cruise ship studied by Vivancos et al.(13) Unfortunately, we are not aware of the inter-zonal airflow between the cabins and the hallways; therefore, for simplicity we assumed it was equal to 5% of the ventilation rate of the cabins.

1.1.3 Inhalation rate of susceptible individuals

The average inhalation rate of humans varies based on their age. Moriarty et al. reported the median ages of the 1045 crewmembers and 2666 passengers in the cruise ship were 36 and 69 years old, respectively(7); therefore, the average age of all passengers and crew in the cruise ship was ~60 years old. We estimated the average inhalation rate of 15.7 m^3/day for passengers and crew of the Diamond Princess Cruise Ship based on their average age and the EPA recommended long-term exposure values for inhalation rates.(14) The inhalation rate of SARS-CoV-2 RNA copies for one susceptible individual in zone *i* ($K_{inhalation,i}$) was estimated based on Equation S5.

$$K_{inhalation,i} = P_{time,i} \times \frac{I_{inhalation}}{V_i} \times \left(1 - R_{facemask,i}\right)^2 \qquad \textbf{Equation S5}$$

Where

$P_{time,i}$: Presence Probability of one susceptible individual in zone *i*
$I_{inhalation}$: Inhalation rate of an adult susceptible individual (m^3/hour)
V_i: Volume of zone *i* (m^3)
$R_{facemask,i}$: SARS-CoV-2 RNA removal efficiency of surgical facemasks in zone *i*

We assumed that before the quarantine started, passengers and crewmembers did not use surgical facemasks (i.e., $R_{facemask,i}$ = 0) and subsequently started using surgical masks during the quarantine only when they were in public area (assumptions for $R_{facemask,i}$ are described in detail in Section 3.8).

1.1.4 Time spent in cabins and public areas

Similar to the daily schedule of passengers on a typical cruise ship used by Zheng et al.,(11) we assumed passengers and crewmembers spent an average of 9 hours per day in their cabins and spent rest of their time in public areas before the quarantine began on February 5. After the start of the quarantine, we assumed the majority of passengers stayed in their cabins all of the time and only contacted with their cabin-mates and the crew who provided daily services. It was reported that the passengers in the interior cabins were allowed to have outdoor air breaks once a day during the quarantine period,(15) but the duration of the breaks was not reported. Therefore, we assumed a daily outdoor air break of one hour for those passengers. We assumed that one crewmember provided necessary services to each cabin for 15 minutes per day, similar to Nicas and Jones assumption for the service time of healthcare workers in healthcare centers per each visit.(1)

1.1.5 Inactivation rate on air, surfaces and hand skin

van Doremalen et al. (16) reported the average half-life of SARS-CoV-2 viable viruses in aerosols as well as on copper, cardboard, stainless steel, and plastic surfaces as approximately 1.0, 0.7, 3.5, 5.7, and 7 hours, respectively. We assumed that the majority of surfaces on the ship that individuals were likely to touch were made of either plastic or stainless steel. Therefore, the half-life of viable SARS-CoV-2 viruses on surfaces was assumed to be ~6 hours. The inactivation rate ($K_{inactivation}$) of the viruses in units of per hour was estimated from Equation S6.

$$K_{inactivation} = \frac{\ln(2)}{T_{half-life} \times \left(1 - R_{disinfection}\right)} \quad \textbf{Equation S6}$$

Where
$T_{half-life}$: Half-life of SARS-COV-2 on media (hour)
$R_{disinfection}$: Disinfection efficiency (just applied to surfaces)

Assumptions for $R_{disinfection}$ are described in more detail in Section 3.8. We are not aware of any study to date that reported the inactivation rate of SARS-CoV-2 viruses on human hands. Therefore, we relied on Xiao et al. and Lei et al. studies on modeling the transmission of MERS-CoV and SARS-COV in a hospital setting and air cabins, respectively. (17, 18) They used an inactivation rate of 0.8 per hour for both coronaviruses on skin, which is similar in magnitude to the inactive rate calculated from the half-life of SARS-CoV-2 on cardboard reported in van Doremalen et al.(16) study (i.e., ~1 per hour).

1.1.6 Transfer efficiencies between surfaces

We considered three transfer efficiencies between surfaces in each zone of the cruise ship to estimate the SARS-CoV-2 surface-to-hand ($K_{StH-transmission,i}$), hand-to-surface ($K_{HtS-transmission,i}$), and hand-to-mucous-membranes ($K_{HTMM-transmission}$) transfer rates using Equations S7-9.

$$K_{HtS-transmission,i} = K_{HS-contact} \times \eta_{HtS-transmission} \times P_{time-active,i} \quad \textbf{Equation S7}$$

$$K_{StH-transmission,i} = K_{HS-contact} \times \eta_{StH-transmission} \times P_{time-active,i} \times \frac{A_{hand}}{A_{zone,i}} \times (1 - R_{hand-wash}) \quad \textbf{Equation S8}$$

$$K_{HTMM-transmission} = K_{HF-contact} \times \eta_{HF-transmission} \times \sum_{i} P_{time-active,i} \times F_{coverage,i} \quad \textbf{Equation S9}$$

Where
$K_{HS-contact}$: Contact rate between hands and surfaces (1/hour)
$\eta_{StH-transmission}$: Transfer efficiency of SARS-CoV-2 from surfaces to hand skin (-)
$\eta_{HtS-transmission}$: Transfer efficiency of SARS-CoV-2 from hand skin to surfaces (-)
$P_{time-active,i}$: Probability of being active in zone *i* for one susceptible individual
A_{hand}: Surface contact area of the bottom of hands (m^2)

$A_{zone,i}$: Surface area of zone *i* (m^2)
$K_{HF-contact}$: Contact rate between hands and face (1/hour)
$\eta_{HF-transmission}$: Transfer efficiency of SARS-CoV-2 between hands and face (-)
$R_{hand-wash}$: Removal effectiveness of hand washing (-)

We are not aware of any measured data on the transfer efficiency of SARS-CoV-2 between surfaces such as hands, surfaces, fomites, and mucous membranes. Therefore, we relied upon the assumptions used for transfer efficiencies of MERS-CoV and SARS-CoV between indoor surfaces in Xiao et al. and Lei et al. studies.(17, 18) Table S1 demonstrates these assumptions.

Table S1. Assumptions used for transfer efficiencies of MERS-CoV and SARS-CoV between surfaces of different materials in indoor environments *(17, 18)*

**Donor surface**	**Acceptor surface**	**Transmission efficiency**	**Microorganism and surface type**
Hand	Porous surface	17%	*Staph. saprophyticus*, from hand to fabric
Hand	Non-porous surface	14%	*Rhinovirus*, from hand to brass doorknob
Hand	Mucous membranes	34%	Bacteriophage PRD-1, from hand to mouth<br>Bacteriophage PRD-1, from hand to lips
Porous surface	Hand	0.3%	Bacteriophage MS-2, from cotton to hand
Non-porous surface	Hand	37%	Bacteriophage MS-2, from stainless steel to hand

Based on the information provided in Table S1, we assumed the surface-to-hand, hand-to-surfaces, and hand-to-mucus-membrane transfer efficiencies are 19%, 16%, and 34%, respectively. We also assumed hand-to-surface and hand-to-face contact rates to be 1.5 (± 0.34) touches per minute(19) and 15.7 (± 11.3) touches per hour(20), respectively. We assumed a hand skin area of ~140 cm^2 based on Göker and Bozkir.(21)

The next section describes key processes and assumptions used in the Markov chain model to track virus injection and transmission across short- and long-range distances on the ship.

1.2 Injection rates of SARS-CoV-2 to various states aboard the cruise ship

Similar to the Markov Chain Matrix, we considered 12 '*states'* for the injection array ($I_k$described in Equation S4) aboard the Diamond Princess Cruise Ship. We assumed that SARS-CoV-2 RNA copies could reach to the "HVAC System" and "Inactivation–Removal" states only from other states, not directly after they are injected from the infectors (i.e. direct injection rate to the HVAC System and Inactivation–Removal states were assumed to be zero). We assumed that infected individuals injected infectious viral particles into the defined spaces onboard the cruise ship in two forms: large diameter droplets and smaller diameter inhalable aerosols. Similar to MCM, we generated a new injection array for every day during the simulation period based on the number of infectors and susceptible individuals, passengers and crew interactions, and type of infection control strategy that was adopted (e.g. wearing facemasks, explored in detail in Section 3).

1.2.1 Injection of SARS-CoV-2 RNA copies in the form of large droplets and inhalable aerosols to the surfaces and indoor air

We assumed SARS-CoV-2 RNA viruses injected in the form of large droplets (i.e., > ~10 µm in diameter, as described in Section 3.4) deposit rapidly onto surfaces of the cruise ship. Viruses are tracked in terms of RNA copies because we utilize the only known available empirical data on SARS-CoV-2 aerosol concentrations and deposition rates in the literature, which report viral RNA

rather than infectious/viable virus, as described in Section 3.3. The injection rate of SARS-CoV-2 RNA copies on surfaces of zone *i* in the form of large droplets per breath ($E_{surface,i}$) was estimated using Equation S10:

$$E_{surface,i} = \frac{N_{infector,i} \times R_{shedding} \times F_{time,i} \times \left(1 - R_{facemask,i}\right)}{N_{breath}} \qquad \textbf{Equation S10}$$

$N_{infector,i}$: Number of infectors in zone *i*
$F_{time,i}$: Time fraction of presence in zone *i*
N_{breath}: Number of breaths per hour

We assumed that before the quarantine started, passengers and crewmembers did not use surgical facemasks (i.e., $R_{facemask,i}$ = 0) and subsequently started using surgical masks during the quarantine only when they were in public area (assumptions for $R_{facemask,i}$ are described in detail in Section 3.8).

The injection rate of SARS-CoV-2 RNA copies in the form of inhalable aerosols (i.e., less than ~10 µm in diameter, based on available data, as explored in Section 3.4) into the indoor air of zone *i* in the cruise ship ($E_{exhalation,i}$) was estimated from Equation S11:

$$E_{exhalation,i} = \frac{N_{infector,i} \times R_{infectious-aerosols} \times F_{time,i} \times \left(1 - R_{facemask,i}\right)}{N_{breath}} \qquad \textbf{Equation S11}$$

1.2.2 Short-range transmission of SARS-CoV-2 RNA copies

We considered three mechanisms for short-range transmission of SARS-CoV-2 between an infector and a susceptible individual, when both are in close proximity to each other, to include:

(i) Direct deposition of large diameter respiratory droplets to the upper respiratory tracts of susceptible individuals (i.e. deposition onto their mucus membranes)
(ii) Direct deposition of respiratory droplets and aerosols to the hands of susceptible individuals, followed by transmission to their upper respiratory tracts via hand-to-mucus-membranes contact
(iii) Inhalation of smaller diameter respiratory aerosols that deposit in the lower respiratory tracts of susceptible individuals

The probability of direct deposition transmission is a function of the probability of being within a short-range contact zone in front of the infector (i.e., 'close contact') and the probability of large droplet deposition to the individuals' mucus membranes. Similarly, the probability of short-range inhalation transmission is a function of the probability of close contact and the probability of inhalation of infectious aerosols in that zone.

To estimate the probability of close contact with infected individuals, the close-range contact area was defined assuming a conical area in front of an infector with the head angle of 60° and length of 3 meters.(2, 22) The probability that a susceptible individual was present within the close-contact cone was estimated based on the proportion of the zone surface area ($A_{zone,i}$) to the projected surface area of the cone on the floor ($A_{close-contact}$), as demonstrated in Figure S3-a. The probabilities of direct injection of infectious droplets to the mucus membrane and hands of a susceptible individual were estimated by dividing the area of mucus membranes (A_{MM}), and

hands (A_{hand}) to the area of the bottom of the air jet cone 1.5 meters in front of the infector ($A_{direct-injection}$), respectively, as demonstrated in Figure S3-b. The dimensions of human facial features were estimated based on Chen et al., which studied the direct deposition and inhalation pathways of respiratory infection transmission using a mathematical model.(22) The combinations of the surface areas of eyes, nose, and mouth was estimated to be approximately 14 cm^2 per person.

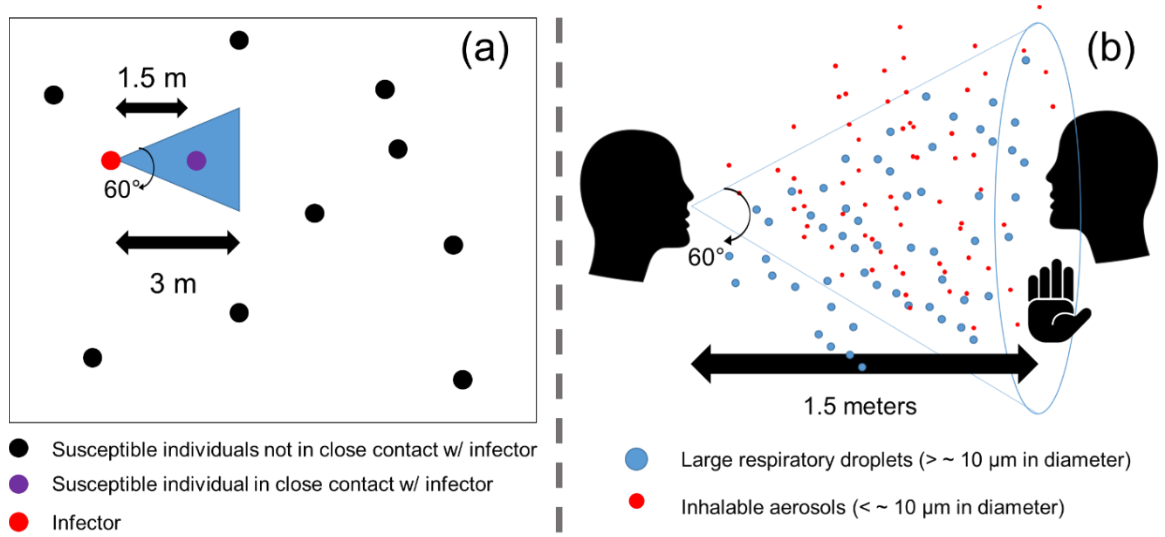

Figure S3. Definition of close-contact zones: (a) the probability of close contact was estimated as the proportion of short-range surface area to the surface area of the indoor zone, and (b) the probability of deposition of infectious droplets on mucus membrane was estimated as the proportion of direct injection area 1.5 meter in front of the infector to the area of target membranes.

The number of SARS-CoV-2 RNA copies deposited *directly* to mucus membranes and the hands of susceptible individuals in each breath by infected individuals ($D_{direct-injection}$) was estimated using Equation S12:

$$D_{direct-injection,i} = N_{infector,i} \times E_{shedding} \times \frac{A_{close-contact}}{A_{zone,i}} \times F_{active-time,i} \times \frac{A_j}{A_{direct-injection}} \times \left(1 - R_{facemask,i}\right) \times \left(1 - F_{coverage,i}\right) \quad \textbf{Equation S12}$$

Where

$E_{shedding}$: Number of SARS-CoV-2 RNA copies injected in large droplets per breath per person
$F_{coverage,i}$: Coverage fraction of facial mucus membrane by facemasks in zone *i*
A_j: Surface area of mucus membranes or the bottom sides of both hands of one susceptible individual

The number of SARS-CoV-2 RNA copies deposited to the lower respiratory tracts of a susceptible individual during close contact with an infector via inhalation ($D_{close-contact-inhalation,i}$) was estimated using Equation S13:

$$D_{close-contact-inhalation,i} = N_{infector,i} \times E_{inhalable-aerosols} \times \frac{A_{close-contact}}{A_{zone,i}} \times F_{active-time,i} \times \frac{V_{inhalation}}{V_{close-contact}} \times \left(1 - R_{facemask,i}\right)^2 \quad \textbf{Equation S13}$$

Where

$E_{inhalable-aerosols}$: Emission rate of SARS-CoV-2 RNA copies in the form of inhalable aerosols per breath per person

$V_{inhalation}$: Volume of inhaled air per breath (m^3)

$V_{close-contact}$: Volume of close contact cone (m^3)

Again, we assumed that before the quarantine started, passengers and crewmembers did not use surgical facemasks (i.e., $R_{facemask,i}$ & $F_{coverage,i}$ = 0) and subsequently started using surgical masks during the quarantine only when they were in public area (assumptions for $R_{facemask,i}$ and $F_{coverage,i}$ are described in detail in Section 3.8).

1.3 Dose Response Model

To estimate the subsequent infection risk of SARS-CoV-2 viruses deposited to different sites of susceptible individuals, we used a negative exponential dose-response model, which implies that a single particle can start an infection and all single particles are independent of each other. The probability of infection for one susceptible individual ($P_{infection}$) in the cruise ship was calculated according to Equation S14:

$$P_{infection} = \frac{Number\ of\ infected\ cases}{Number\ of\ Susceptibles} = 1 - \exp[-(\alpha_{URT} \times N_{URT} + \alpha_{LRT} \times N_{LRT})] \quad \textbf{Equation S14}$$

Where

N_{URT} and N_{LRT}: Number of viable SARS-CoV-2 RNA copies in upper and lower respiratory tracts of one susceptible individual

α_{URT} and α_{LRT}: Infectivity of SARS-CoV-2 for upper and lower respiratory tracts

The 50% infection dose (ID_{50}), or the number of viruses necessary to infect a susceptible individual in 50% of a sample population, of the SARS-CoV-2 for upper and lower respiratory tracts can be estimated from Equation S15(1, 20):

$$ID_{50} = \frac{\ln(2)}{\alpha} \qquad Where \quad ID_{50} \geq \ln(2) \quad \textbf{Equation S15}$$

Estimates of ID_{50} and infectivity for upper and lower respiratory tracts (URT and LRT) play a critical role in understanding the transmission of airborne infectious diseases. However, we are not aware of any clinical studies to date that report these values for SARS-CoV-2 in humans or animals.

Therefore, we rely on our model approach to back-calculate effective ID_{50} for upper and lower respiratory tracts (on a basis of RNA copies) by the following steps:

1- We selected an effective reproduction number for the COVID-19 outbreak on the cruise ship during the presence of the index case in the range of 1 and 6 based on Mizumoto and Chowell,(23)
2- We estimated the probability of infection based on the number of infected cases and susceptible individuals during the presence of the index case onboard the cruise ship,
3- We estimated the average number of SARS-CoV-2 viruses (i.e. RNA copies) that reached to the upper and lower respiratory tracts of the susceptible individuals during the first period of the analysis, and
4- We defined three scenarios for the proportion of infection dose of URT to LRT (i.e. ID_{50} URT/LRT = 1, 10, 100) and back-calculated the infection dose accordingly (explained in more detail in Section 3.7).

Step 4 allows us to test three logarithmically spaced assumptions for the ratio between ID_{50} for URT and LRT without knowing (or needing to know) the actual magnitude of ID_{50}, while also anchoring ID_{50} to RNA copies rather than infectious units (e.g., PFU). Thus, the utility of the model approach goes beyond estimating transmission modes; the framework also allows for some inference of these important parameters by identifying which model scenarios and assumptions most accurately fit cumulative case counts.

1.4 Contribution from each transmission mode

In addition to estimating the number of infected cases with the model framework, we also estimated the contribution of several infection transmission modes, including direct deposition of droplets, fomite, and both close- and long-range inhalation of aerosols, to the estimated number of infected cases in both cabins and public areas using Equation S16:

$$C_{infection,k,r,p} = \sum_{l=0}^{D_p} \left\{ \frac{N_{infected,r,l}}{N_{infected,total,p}} \times \frac{1 - \exp\left(-N_{virus,k,r,l} \times \alpha_k\right)}{\sum_{k,r}\left[1 - \exp\left(-N_{virus,k,r,l} \times \alpha_k\right)\right]} \right\} \qquad \textbf{Equation S16}$$

Where

k: Four considered scenarios for infection transmission modes including direct deposition, fomite, long-range inhalation, and short-range inhalation

r: Two considered micro-environments in the cruise ship including cabins and public areas

p: Three considered simulation periods including during the whole outbreak, before the passenger quarantine began, and after the passenger quarantine began

$C_{infection,k,r,p}$: Infection contribution associated with transmission mode k in microenvironment r in simulation period p

D_p: Number of simulation days in simulation period p, which was considered to be 36, 16, and 20 for total duration of the outbreak before all passengers disembarked, before the passenger quarantine began, and after the passenger quarantine began, respectively

$N_{infected,r,l}$: Number of infected cases in microenvironment r on day l of the simulation period

$N_{infected,total,p}$: Total number of infected cases in the cruise ship during the simulation period p

$N_{virus,k,r,l}$: Number of SARS-CoV-2 RNA copies that reached the target respiratory tract (i.e. LRT for inhalation and URT for direct deposition and fomite) via transmission mode k in microenvironment r on day l of the simulation period

α_k: Infectivity of SARS-CoV-2 for the target respiratory tract (i.e. LRT for inhalation and URT for direct deposition and fomite)

Direct deposition transmission was assumed to occur only when susceptible individuals were within the close-range contact area (defined in Section 1.2.2) and subject to direct contact with respiratory droplets from infected individuals (defined in Section 3.4). Short- and long-range inhalation transmission was assumed to occur via inhalation of aerosols either inside or outside the close-contact area, respectively. Fomite transmission was assumed to occur when susceptible individuals came in contact with contaminated surfaces, which could be contaminated by infected individuals through direct touching, direct deposition of respiratory droplets, and/or deposition of respiratory aerosols at any time point in the model framework. This approach also allows for summarizing infection contributions only by transmission mode, contact-range, micro-environment, or simulation period independently, as needed.

2. Combining the Complex Infection Transmission Risk Model with a Developed Epidemic Model

Generally, infection transmission risk models have been applied to the outbreaks during less-than-a-day events. In these events, the number of infectors and susceptible individuals were assumed to stay constant during the outbreak. However, the COVID-19 outbreak in the Diamond Princess Cruise Ship was a unique situation, where the outbreak happened over a 40-day period. Therefore, we combined the complex infection transmission model with a newly developed version of the Reed-Frost epidemic model to simulate the transmission of COVID-19 in the Diamond Princess Cruise ship.

2.1 Developing an adjusted Reed-Frost model for the cruise ship outbreak

The Reed-Frost model is one of the simplest stochastic epidemic models. The model has been used for estimating the transmission of infectious diseases such as tuberculous(24) and measles,(25) although in some of those studies (e.g. Chen et al.(25)) it was shown that the model alone cannot explain the infection transmission within the studied indoor environment. As the basic Reed-Frost model that was described by Abbey(26) could not be used for this modeling work, we developed an advanced version of the Reed-Frost model for the cruise ship outbreak. The assumptions of the newly developed Reed-Frost model are described in the following:

(i) The infection is spread from infected individuals to others by four main transmission pathways including long-range inhalation, short-range inhalation, direct deposition, and fomite,
(ii) A portion of susceptible individuals in the group, after such contact with an infectious person in a given period, will develop the infection and will be infectious to others (the portion of '*susceptibles'* who will develop the infection is estimated by the complex transmission risk model),
(iii) The probability of coming into adequate contact with any other specified individual in the group within one time interval depends on the interaction behavior of the individual and is estimated using the Markov chain method,

(iv) The susceptible individuals in the cruise ship were isolated from others outside the cruise ship, and
(v) These conditions remain constant during one-whole day of the outbreak.

For the epidemic model, we took the following steps:

1- The numbers of infected cases among susceptible individuals, some of whom were cabinmates with infected individuals and some were not (described in Section 1.1), were estimated using the complex transmission risk model at the end of each simulation day.

2- The infected cases were assumed to develop infection and become **infectors** after the latent period, which was estimated by reducing the assumed sub-clinical infectious period from the incubation period.

3- The number of cabins with at least one infected individual (i.e. 'infected cabins') was calculated at the end of each simulation day by assuming the number of newly infected cabins is equal to the number of newly infected cases who were not in one of the previously infected cabins at the beginning of the simulation day.

4- The numbers of susceptible individuals who were not cabinmates with an infector ($N_{susceptibles-common}$) and susceptible individuals inside the infected cabins ($N_{susceptibles-cabin}$) at the beginning of each simulation day (d) were estimated using the Equations S17-18 (except for the first period of infection transmission in the cruise ship):

$$N_{susceptibles-common}(d) = N_{total-onboard} - \left[N_{infected-cabin}(d) \times N_{average-cabin}\right]$$ **Equation S17**

$$N_{susceptibles-cabin}(d) = \left[N_{infected-cabin}(d) \times N_{average-cabin}\right] - N_{infector}(d) - \sum_{i=0}^{d-1} N_{detected-cases}(i)$$ **Equation S18**

Where

$N_{total-onboard}$: Total number of passengers and crew onboard (constant during the outbreak)

$N_{infected-cabin}$: Estimated number of infected cabins at the beginning of each day

$N_{average-cabin}$: Average number of individuals in one cabin

$N_{infector}$: Number of infectors

$\sum N_{detected-cases}$: Cumulative number of detected infected cases or disembarked individuals from the cruise ship

5- We assumed the infected cases could spread infectious particles only one day after the incubation period, when their clinical symptoms began.

2.2 Epidemic characteristics of the outbreak during four assumed infection transmission periods

As mentioned previously, we divided the transmission patterns of SARS-CoV-2 on the Diamond Princess Cruise Ship into four periods. Each of these periods had different epidemic characteristics that we considered in our model.

During the first period, when the only infector onboard the cruise ship was the index case, we assumed no one was sharing a cabin with the index case, as none of the sources reported any information regarding the index case cabinmate.(7, 15) Therefore, the number of susceptible individuals in the infected cabins was zero. The number of susceptible individuals in the public area during this period was assumed to be 3,188 (i.e. 2,666 guests and 522 crewmembers). It is noticeable that the total number of crewmembers in the cruise ship on February 4th, when the quarantine started, was 1045, but most likely not all of them were interacting with passengers or would have been able to be in the same indoor environment with the index case (e.g., crewmembers who worked in the engine, kitchen, or control areas). With a lack of reliable sources on the number of crewmembers interacting with passengers during the first period, we simply assumed that half of the crewmembers possibly interacted with any passengers or were able to be in the passenger-accessible areas during the first period. The number of susceptible individuals among the infectors' cabin mates and other individuals during the other infection transmission periods were calculated from the aforementioned equations.

We assumed that after the passenger quarantine began, most of the passengers spent their time in their cabins, except the passengers in interior cabins, who were allowed to have outdoor time in open public areas under the guidance of Japanese Ministry of Health.(15) As the Diamond Princess Cruise Ship had 377 interior cabins(8) and the average number of passengers in each cabin was 1.98 people,(7) we assumed that 754 passengers in the interior cabins used the public areas during the quarantine for one hour per day. We also assumed that during the quarantine the indoor public areas were closed and only half of the crewmembers (i.e., 523 crewmembers) on the cruise ship were considered as '*essential'* crew and interacted with other crewmembers and passengers in the hallways and areas such as the kitchen, control rooms, and the health clinic to provide necessary services. The rest of the crewmembers were assumed to remain in their interior cabins and to have followed similar guidelines as the passengers.

2.3 Adding checkpoint conditions to the epidemic model

We introduced several checkpoint conditions to the model to avoid unreasonable outcomes, as follows:

1- The cumulative number of infected cases and infectors should be always smaller than the total number of people on board.
2- The number of infectors and susceptible individuals should not be negative.
3- The number of infected cabins should be less than the total number of cabins in the cruise ship.
4- The sum of the number of disembarked and onboard infectors should remain lower than the total number of people onboard at the beginning of the quarantine.
5- The sum of the number of disembarked and onboard infectors should remain lower than number of infected rooms times the average number of cabinmates in the cruise ship (i.e. ~2)

3. Identifying the primary unknown or uncertain epidemic and infection transmission modeling parameters

Using these models requires numerous assumptions or estimates for unknown or uncertain input parameters, which were culled from existing literature where possible and otherwise estimated or assumed using known information about the Diamond Princess Cruise Ship. Because there is high uncertainty around several critical model parameters, especially those related to COVID-19 epidemic and mechanistic transmission characteristics, the interactions among individuals onboard, and the effectiveness of infection control strategies adopted during the quarantine period, we utilized a scenario modeling approach in which values for unknown or uncertain epidemic and transmission modeling parameters were varied over a wide range of possibilities to generate a matrix of possible solutions. This approach resulted in a total of 21,600 model iterations, with daily case counts and daily cumulative case counts among passengers aboard the ship serving as the primary model outcomes. Only those model scenarios with an acceptable coefficient of determination (R^2) between reported and modeled daily (i.e., daily $R^2 > 0$) and daily cumulative (i.e., $R^2 > 0.95$) case counts were analyzed further to determine the resulting bounds of these unknown or uncertain parameters. We also performed a sensitivity analysis of several important model parameters (discussed in Section 4).

The utility of this approach is that it allowed us to consider a wide variety of possible input parameters, based in part on emerging empirical evidence in the literature and in part on assumptions from other literature, to seek the most plausible solutions that fit the daily cumulative case numbers, which in turn provide insight into the values of input parameters that were associated with successful model runs. In other words, by selecting a range of unknown or uncertain values of key model input parameters and analyzing only those model results that meet acceptable criteria for predicted cases over time, not only do we infer information on the likely modes of transmission, but we also infer information on the likely ranges or bounds of the original unknown or uncertain input parameters.

Eight key model input parameters with unknown or uncertain values included:

1. Effective incubation period – the time span between infection and detection among infected cases
2. Effective subclinical infectious period – the time span between the onset of an individual's infectious period and the appearance of clinical signs of disease
3. Viral generation rate in aerosols and droplets
4. Viral generation rate among asymptomatic and symptomatic individuals
5. Close interaction frequency
6. Median infectious dose
7. Effective reproduction number for the index case
8. Efficacy of infection control strategies during the quarantine period

3.1 Effective incubation period

We define the effective incubation period as the time span between infection and detection among infected cases. Based on the information available on the COVID-19 outbreak on the Diamond Princess Cruise Ship, the first 10 infected cases demonstrated COVID-19-related symptoms on February 4th, 15 days after the index case boarded the Cruise Ship. A day after that, the quarantine period began, and the majority of passengers and crewmembers had to stay in their cabins. During the quarantine, suspected passengers and crew were tested for COVID-19 infection on a daily basis, and positive cases were then physically separated from other

passengers and crewmembers by sending them to local hospitals.(10, 27) Laboratory tests by PCR were conducted in the cruise ship focusing on *symptomatic cases*, especially at the early phase of the quarantine.(23) Therefore, we assumed that infectors, except the index case, infected the susceptible individuals in the cruise ship until they tested positive for COVID-19 during the quarantine period. We also assumed that it took one day for the laboratory to send back the results.

Lauer et al. estimated that 97.5% of infected cases who develop symptoms will do so within 11.5 days (95% CI: 8.2 to 15.6 days) of infection.(28) They also estimated the median incubation period (i.e., the time span before clinical signs show) was 5.1 days (95% CI: 4.5 to 5.8 days) among 181 confirmed cases.(28) Another study estimated the mean incubation period to be 5.2 days (95% CI: 4.1 to 7.0), with the 95th percentile of the distribution at 12.5 days among patients with a median age of 59 years.(29) As the detection of the infected cases in the cruise ship was mostly based on symptomatic cases at the beginning of the quarantine and gradually all passengers and crew were tested for COVID-19 infection toward the end of the quarantine, we assumed that the average time span between infection and detection of infected cases (i.e. effective incubation period) in the cruise ship was anywhere between 6 and 15 days. Thus, we varied model inputs for this parameter from 6 to 15 days in increments of 1 day. This assumption was not free of limitations, particularly for asymptomatic cases.

3.2 Effective subclinical infectious period for infectors in the cruise ship

Another critical parameter for the epidemic modeling is the time span between the onset of the infectious period and the appearance of clinical signs of disease, which is also known as subclinical infectious period.(30) A positive subclinical infectious period demonstrates the time when infected cases could spread pathogens of an infectious disease before the clinical signs appear. Several studies suggested that subclinical infectious period is a key area of uncertainty and should be considered into account in infection transmission models.(31, 32) A description a family cluster of SARS-CoV-2 infection involving 11 patients in Nanjing, China, demonstrated that transmission of the COVID-19 can occur as early as five days before onset of symptoms.(33)

Similar to the effective incubation period, because of the unique situation of the outbreak in the Diamond Princess Cruise Ship, we assumed an *effective subclinical infectious period* between 1 and 5 days for the infected cases in the cruise ship, with model inputs ranging from 1 to 5 days in increments of 1 day. The effective subclinical infectious period demonstrates on average how many days before the infected cases tested positive they spread the SARS-CoV-2 in the cruise ship.

3.3 Emission rate of SARS-CoV-2 from symptomatic and asymptomatic (or pre-symptomatic) cases

We considered the plausibility of different emission rates of SARS-CoV-2 from both symptomatic and asymptomatic (or pre-symptomatic) cases. We estimated the proportion of asymptomatic (or pre-symptomatic) cases to symptomatic cases based on the reported number of asymptomatic and symptomatic cases on the Diamond Princess Cruise Ship between February 5 and 20 provided in Mizumoto et al.(23) For our purposes, asymptomatic and pre-symptomatic are interchangeable because an individual classified as asymptomatic could have also been pre-symptomatic if they simply developed symptoms later than the simulation time period.

There is very limited information in the current literature on the emission rate of SARS-CoV-2 from asymptomatic (or pre-symptomatic) and symptomatic cases. Chia et al. measured the

concentration of SARS-CoV-2 in two hospital rooms with symptomatic and asymptomatic patients.(34) The airborne concentrations of SARS-CoV-2 in the asymptomatic and symptomatic patient rooms were 1843 and 3384 RNA copies m^{-3}, respectively.(34) In another study, Arons et al. concluded that asymptomatic and pre-symptomatic residents in a nursing facility had the potential for substantial viral shedding, although they were unable to quantify their contributions in transmission of SARS-CoV-2 in the facility.(35) They also reported similar viral loads for asymptomatic, pre-symptomatic, and typical and atypical symptomatic individuals.(35)

Therefore, to consider a range of plausible scenarios involving asymptomatic (or pre-symptomatic) and symptomatic cases, we considered two scenarios for the ratio of SARS-CoV-2 emission rates of asymptomatic to symptomatic cases, including 0.545 and 1 based on Chia et al.(34) and Arons et al.(35), respectively. This remains a highly uncertain parameter, but our selection of inputs that span a factor of two is intended to capture a relatively wide range in this parameter and will ideally offer insight into the most likely ratio based on model outcomes.

3.4 Emission rate of SARS-CoV-2 RNA copies in the form of large droplets and inhalable aerosols

We considered the emission rate of large respiratory droplets and small inhalable aerosols separately. To evaluate the transmission modes SARS-CoV-2, we needed to estimate the emission rate of SARS-CoV-2 RNA copies in the form of large droplets (> ~10 µm in diameter) and inhalable aerosols (≤ ~10 µm in diameter). The size of particles containing infectious viruses has a direct impact on the viral transmission mechanisms in indoor environments. Moreover, we assumed that only inhalable aerosols (≤ ~10 µm in diameter) can reach to the lower respiratory tracts of susceptible individuals, similar to prior assumptions by Nicas and Jones in their estimates of the relative contribution of four transmission pathways of influenza viruses in a typical health care center.(1) We defined three scenarios for emission rates based on two studies that measured size-resolved concentrations of SARS-CoV-2 RNA copies in patient rooms, as described below.

3.4.1 Measurements of SARS-CoV-2 RNA copies in patient areas in Fangcang and Renmin hospitals in Wuhan, China

The first scenario was based on Liu et al., which measured the number of SARS-CoV-2 RNA copies deposited on two locations on the floor of a 16-m^2 ICU room in Renmin Hospital, Wuhan, China and concentration of aerosols smaller than 10 µm in nine patient areas in *Fangcang* and Renmin hospitals in Wuhan, China.(36) The normalized deposition rate of SARS-CoV-2 in the ICU rooms were 31 copies m^{-2} $hour^{-1}$ and 113 copies m^{-2} $hour^{-1}$ for a location under medical equipment 2 meters from a patient's bed with severe symptoms and another location 3 meters from the same patient's bed without any objects above it, respectively. As the ICU room was relatively small and most probably well-ventilated with a high ventilation rate, we assumed that these measurements occurred under well-mixed conditions. Therefore, we estimated the emission rate of large droplets (> 10 µm in diameter) that rapidly deposit onto the floor (i.e. *shedding* rate: $R_{shedding}$) to be between 496 and 1808 RNA copies per hour per person, with the best estimate of 1152 (i.e. [(113 + 31 copies m^{-2} $hour^{-1}$) / 2] × 16 m^2) RNA copies per hour per person. This is the only study to date of which we are aware that empirically assessed SARS-CoV-2 deposition rates.

The same study also measured airborne SARS-CoV-2 concentrations in several different sites. The measurements of airborne SARS-CoV-2 concentrations in "patient areas" were conducted in three workstation zones, one patient mobile toilet room, one ICU, one CCU, and one ward zone. The floor area of the workstation zones and the toilet room in the *Fangcang* hospital were

approximately 500 m^2 and 1 m^2, respectively. The toilet room did not have any ventilation and the workstation zones were relied on natural ventilation only. The measured airborne concentrations of SARS-CoV-2 varied between 0 and 9 copies m^{-3} in the workstation areas and was 19 copies m^{-3} in the toilet room. The airborne concentration of SARS-CoV-2 was zero in the other patient areas including the ICU, CCU, and ward zones of the Renmin Hospital. Moreover, the number of patients in the workstation areas was between 100 and 200 people. Patients in the workstation zones had mild symptoms, while the patients in the ICU and CCU rooms had severe symptoms. We use these concentrations in Section 3.4.3 to back-calculate emission rates.

3.4.2 Size-resolved measurements of SARS-CoV-2 RNA copies in patient rooms at the National Centre for Infectious Diseases in Singapore

Our second and third scenarios for the SARS-CoV-2 emission rates – or more accurately, the ratio between large droplet and inhalable aerosol emissions – were based on Chia et al., which conducted bioaerosol sampling in three airborne infection isolation rooms at the National Centre for Infectious Diseases, Singapore.(34) The rooms had mechanical ventilation systems delivering 12 air changes per hour, an average temperature of 23°C, relative humidity of 53 – 59%, and exhaust flow of 579.6 m^3/h. They deployed six NIOSH BC 251 bio-aerosol samplers in each room in the general ward to collect air samples. Particles collected with the NIOSH sampler were distributed into three size fractions including particles >4 μm, 1-4 μm, and <1 μm in diameter.

Among the three hospital rooms where patients were treated, no airborne SARS-CoV-2 RNA copy was detected in the room with a patient on 9th day of illness. The other two patients were on their 5th day of illness, and one of them was symptomatic and the other one was asymptomatic. The airborne SARS-CoV-2 concentration in the symptomatic patient room was 2000 and 1384 copies per m^3 for particles >4 μm, 1-4 μm in diameter, respectively and in the asymptomatic patient room was 927 and 916 copies per m^3 for particles >4 μm, 1-4 μm in diameter, respectively. No virus was detected in the <1 μm size ranges, although the authors mention that this could be due to low viral extraction efficiencies from the filter used on this stage (the other two stages deliver bioaerosols directly into fluid media for analysis). Regardless, this work considers only the 1-4 μm and <4 μm results from this study.

Because we consider ~10 μm as the cut off size between large droplets and inhalable aerosols (which we recognize is a somewhat arbitrary, albeit practical and useful, definition), we defined two scenarios based on the measurements from Chia et al. In one scenario, we assumed that half of the SARS-CoV-2 RNA copies collected in particles >4 μm in diameter were between 4 and 10 μm in diameter and the rest were in particles >10 μm in diameter (the NIOSH sampler does not have an upper size cut-off to our knowledge). In the other scenario, we assumed that half of the total measured SARS-CoV-2 RNA copies were in particles ≤10 μm and the other half were in particles >10 μm in diameter, primarily for simplicity and to provide another plausible value for this input parameter.

3.4.3 Applying well-mixed number balance model to estimate the emission rates of SARS-CoV-2 RNA copies in forms of large droplets and inhalable aerosols

We assumed steady-state well-mixed conditions for the workstation zones and the mobile toilet room in Liu et al.(36) and in the airborne infection isolation rooms in Chia et al. (34) to back-calculate emission rates for inhalable aerosols. For all of the rooms, we assumed that the resuspension rate is negligible compared to other transmission mechanisms of SARS-CoV-2. The emission rate of SARS-CoV-2 RNA copies in the form of inhalable aerosols smaller than ~10 μm in diameter ($E_{inhalable-aerosols}$, copies per hour per person) was estimated using Equation S19:

$$E_{inhalable-aerosols} = \frac{C \times V \times (K_{deposition} + \lambda_{ventilation})}{N_{patient}}$$ **Equation S19**

Where

C: Steady-state concentration of infectious aerosols (copy per m^3)
V: Volume of the room (m^3)
$N_{patient}$: Number of patients in the room (person)
$\lambda_{ventilation}$: Air exchange rate due ventilation (per hour)
$K_{deposition}$: Deposition rate (per hour)

Patient area characteristics of *Fangcang* Hospital, Wuhan, China: Information related to the ventilation rate in the *Fangcang* Hospital was limited. A total of 16 temporary "shelter hospitals," or *fangcang* hospitals, were built in Wuhan, China to treat mild COVID-19 cases. Based on the information provided in Liu et al. (36) regarding the various workstation zones, each with a surface area of ~500 m^2, we believe the measurements were likely conducted in Wuhan Sports Center Gymnasium, Hubei province, China, which was one of the or *fangcang* hospitals.(37, 38) We know that the workstations relied on natural ventilation only. As the *Fangcang* Hospital was built from a converted sports arena before the outbreak started, we assumed a typical air exchange rate between 0.15 and 3.5 per hour with an average air exchange rate of 1.8 per hour for the hospital.(39) The volume of the workstations was estimated by multiplying their floor area to the assumed ceiling height of hospital (approximately 10 meters), which was estimated using photographs of the arena. For the number of patients in the measured areas, Liu et al. reported the number of patients in each workstation varied between <100 and >200 patients.(36) We assumed that one person used the mobile toilets at a time and it was occupied half of them time.

Patient room characteristics of National Centre for Infectious Diseases, Singapore: As Chia et al.(34) reported, the isolation rooms had mechanical ventilation delivering 12 air changes per hour and an exhaust flow of 579.6 m^3/h. Therefore, the volume of each room was estimated to be around 48 m^3. There was only one patient in each room.

Deposition rate of inhalable aerosols: Liu et al. measured size resolved distributions of SARS-CoV-2 RNA copies in aerosols less than 10 µm in diameter in three locations in the *Fangcang* Hospital, Wuhan, China. We calculated the average proportion of SARS-CoV-2 RNA copies in the five particle size bins reported in the study.(36) Next, we mapped the size distribution of SARS-CoV-2 RNA copies on the size-resolve distribution of particle deposition rate reported in Riley et al. (40) to estimate the bulk deposition rate of SARS-CoV-2 RNA copies in aerosols, which was estimated to be about 0.64 per hour. A similar approach was used previously in Azimi and Stephens (41) for estimating the bulk deposition rate of influenza viruses. Table S2 demonstrates the relative size distribution of SARS-CoV-2 RNA copies, the estimated deposition rate for each particle size bin, and the estimated bulk deposition rate for SARS-CoV-2 RNA copies in inhalable aerosols less than 10 µm.

Table S2. Estimates of size-resolved and bulk deposition rates of SARS-CoV-2 RNA copies in inhalable aerosols less than 10 μm in diameter

**Particle Size Range (D_p, μm)**	**Geo-mean (μm)**	**Deposition Rate for D_p[a] (1/hr)**	**Average Relative Distribution of SARS-CoV-2 RNA[b]**	**SARS-CoV-2 Aerosol Deposition Rate (1/hr)**
0.01 - 0.25	0.05	0.072	10.0%	0.007
0.25 - 0.5	0.35	0.068	39.2%	0.027
0.5 - 1.0	0.71	0.108	18.3%	0.020
1.0 - 2.5	1.58	0.378	6.6%	0.025
2.5 - 10	5.00	2.160	25.8%	0.557
Bulk deposition rate of SARS-CoV-2 RNA copies in inhalable aerosols less than 10 μm in diameter				0.636

[a] Calculated from Riley et al. (40); [b] Liu at al.(36)

Table S3 demonstrates the assumptions for estimating the emission rate of SARS-CoV-2 RNA copies in the form of inhalable aerosols ($E_{inhalable-aerosols}$,) using Equation S19 based on Liu et al.(36) and Chia et al.(34)

Table S3. Estimating the emission rate of SARS-CoV-2 RNA copies in the form of inhalable aerosols using Equation S19 parameters

	Measurement Location	V (m^3)	$N_{patient}$	$K_{deposition}$ (1/hr) [d]	$\lambda_{ventilation}$ (1/hr)	C (copy/m^3)	$E_{inhalable-aerosols}$ (copy / hr /person)
Liu et al. (36)	Fangcang hospital Zone A-I (TSP)	5000[a]	200	0.64	1.8[e]	1	61
	Fangcang hospital Zone A-II (TSP)	5000[a]	100	0.64	1.8[e]	9	1098
	Fangcang hospital Zone B (TSP)	5000[a]	200	0.64	1.8[e]	1	61
	Fangcang hospital Zone C (TSP)	5000[a]	200	0.64	1.8[e]	5	305
	Fangcang hospital mobile toilet (TSP)	2[b]	0.5	0.64	0	19	48.64
Chia et al.(34)	Symptomatic patient room (>4 μm)	48.3[c]	1	0.64	12	2000	1,221,024
	Symptomatic patient room (1-4 μm)	48.3[c]	1	0.64	12	1384	844,949
	Asymptomatic patient room (>4 μm)	48.3[c]	1	0.64	12	927	565,945
	Asymptomatic patient room (1-4 μm)	48.3[c]	1	0.64	12	916	559,229

TSP: Total suspended particles with no upper size limit
[a] Floor area of 500 m^2 times assumed ceiling height of 10 m
[b] Floor area of 1 m^2 times assumed height of 1 m
[c] Exhaust rate of 579.6 m^3/hr divided by air exchange rate of 12 per hour
[d] Described in Table S2
[e] Assumption based on the air exchange rate of a typical sport area(39)

Based on the estimates of the deposition rate and patient area characteristics, we estimated the emission rate of SARS-CoV-2 RNA copies in inhalable aerosols smaller than 10 μm in diameter to be ~315 RNA copies per hour, with a likely range between ~50 and ~1100 copies per hour for the *Fangcang* Hospital scenario (Emission Scenario 1). Similarly, the emission rate of SARS-CoV-2 RNA copies in the form of inhalable aerosols (≤10 μm in diameter) and large droplets (>10 μm) in isolation patent rooms in the National Centre for Infectious Diseases in Singapore was

estimated to be 1,455,461 and 610,512 copies per hour per person in Emission Scenario 2, respectively, and 1,032,986 copies per hour per person for both inhalable particles and large droplets in Emission Scenario 3.

It is worth noting that our estimation of SARS-CoV-2 RNA copy exhalation rates are within the realm of plausibility in comparison to other studies that have measured the number of RNA copies of other respiratory pathogens in the exhaled breath. For example, Milton et al. measured the median Influenza virus copy number in aerosol particles (< 5 µm) exhaled by patients equal to ~200 copies per hour with 1st and 3rd quartiles of ~80 and ~400 copies per hour and a range from lower than detection level to 2.6 x 10^5 copies per hour.(42) In another study with a similar approach, Yan et al. measured the geometric mean of emission rate of influenza virus RNA copies in exhaled fine aerosols (< 5 µm) equal to 7.6 x 10^4 copies per hour with a range from not detectable to 8.8 x 10^7 copies per hour.(43) It is also worth noting that this model was designed to back-calculate 50% infective dose for upper and lower respiratory tracts based on our assumptions for emission rate of SARS-CoV-2 RNA copies (Section 1.3); therefore, the absolute magnitude of these values are not crucial in the model, but rather the ratio between large respiratory droplets and small inhalable aerosols is most useful.

3.5 Minimum close-range interaction time in the cabins

Close-range interactions between people plays a critical role in the transmission of infectious airborne diseases. In this model, we defined a close-range interaction as any interaction between an infector and a susceptible individual that happens within a 3-meters-length hypothetical zone in front of the infector, as described in Section 1.2.2. In the public areas aboard the Diamond Princess Cruise Ship, we assumed that infectors and susceptible individuals interact with each other randomly. However, to estimate the close interaction time inside the cabins, particularly after the passenger quarantine was enacted, we considered two scenarios. In the “Standard” scenario, we assumed that the infector and susceptible individual had a minimum close interaction of 8 hours; in the “Extended” scenario, we assumed that the infector and susceptible individual had a minimum close interaction of 16 hours. We assumed that the passengers spent 9 hours on average in their cabins before the quarantine started; therefore, in the “Extended” scenario, passengers’ close interaction time in cabins before the quarantine began was limited to 9 hours.

3.6 COVID-19 effective reproduction number for the index case

The effective reproduction number is defined as the average number of secondary cases per infectious cases in a population. Estimating the effective reproduction number of COVID-19 on the Diamond Princess Cruise Ship has been used for investigating the result of interventions strategies that were imposed on travelers and crew aboard the cruise ship.(23) Herein, we used the estimates of COVID-19 effective reproduction number for the index case to back-calculate the ID_{50} and infectivity of SARS-CoV-2 for upper and lower respiratory tracts as describe in detail in Section 1.3. For the effective reproduction number, we relied on Mizumoto and Chowell, which estimated the range of effective reproduction number to be between 1 and 6 during the five days that the index case was onboard the cruise ship.(23)

3.7 SARS-CoV-2 median infectious dose

Our knowledge on the transmission mechanisms of SARS-CoV-2 is still very limited. For example, still there is no information about the 50% infective dose (ID_{50}) of SARS-CoV-2 for upper and lower respiratory tracts (URT and LRT).(44) Moreover, the proportions of SARS-CoV-2 depositing in the LRT and URT of a susceptible individual when they inhale infectious aerosols are not characterized. To circumvent this gap, we (1) assumed different effective ID_{50} of SARS-CoV-2 for

(i) inhalation and (ii) fomite and direct deposition transmission pathways, and called them ID_{50} for (i) LRT and (ii) URT, respectively, and (2) estimated the ID_{50} of SARS-CoV-2 for upper and lower respiratory tracts by (i) using estimates of the effective reproduction number of COVID-19 in the Diamond Princess Cruise Ship from the existing literature during the time that the index case was onboard the cruise ship (Section 3.6); and (ii) considering a variety of scenarios for the proportion of ID_{50} of SARS-CoV-2 for URT to the LRT (i.e. 1:1, 10:1, and 100:1). In Section 1.3, we explained our approach for estimating the ID_{50} of SARS-CoV-2 in more detail. Here, we describe how we arrived at these three logarithmically spaced assumptions.

In this model, we estimated the number of virus copies that were predicted to reach the upper and lower respiratory tracts of susceptible individuals separately because the site and efficiency of respiratory deposition would be different for different droplet sizes. For example, larger droplets would deposit more efficiently in the upper respiratory tract(45), while the pathogens carried by inhalable aerosols (≤ 10 µm in diameter) would be able to reach to the lower respiratory tract.(46) It is also shown that for some viral airborne diseases such influenza the infectivity of viruses reaching to LRTs of an susceptible individual is about two orders of magnitude higher than the viruses deposited in the URTs(47) (i.e. influenza human ID_{50} is estimated between 0.6 and 3 TCID50 for lower respiratory tracts(48) and between 127 and 320 TCID50 for upper respiratory tracts(49)). Therefore, our three assumed ranges of the ratio between ID_{50} for URT and LRT of 1, 10, and 100 represent three theoretically possible values across a wide range in magnitude.

3.8 Efficacy of infection control strategies during the quarantine period

Based on the information provided in various sources, we know that the Diamond Princess Cruise Ship was quarantined at Yokohama on February 3rd, 2020, and then a quarantine of all passengers began on February 5th, 2020.(7, 15, 23, 50) During the quarantine, the passengers and crew had to follow strict guidelines, including staying in their cabins all of the time, except for individuals who were in interior cabins, as well as essential crew, and all passengers and crew in the public area had to use facemasks. However, the effectiveness of wearing facemasks for reducing COVID-19 transmission risk in the cruise ship was not measured or reported; therefore, we relied on other studies that have reported particle/viral removal efficiencies of facemasks. We also believe it is reasonable to assume people during the quarantine washed their hand more frequently and effectively amid the safety guidelines that was provided for passengers and crew.

Therefore, we considered two scenarios for the efficacy of infection control strategies adopted on the cruise ship. In the “moderate efficacy” scenario, we assumed that before the passenger quarantine began, the viral removal effectiveness of hand washing ($R_{hand-wash}$) was ~50% by considering a 70% probability of washing hands after touching fomite and a 72.5% viral removal efficiency for washing hands similar to the values used for a typical cruise ship model in Zhang et al.(12) Then, we assumed the viral removal effectiveness of hand washing increased to ~80% after the passenger quarantine began (i.e. probability and viral removal efficiency of 90% for washing hands). We also assumed that before the quarantine, complete surface disinfection was performed on a daily basis at the end of the day. We assumed that after the quarantine started, in addition to the complete end-of-day disinfection, the public area and cabin surfaces were disinfected by crewmembers and passengers several times during the day. Unfortunately, we are not aware of how often the regularly-touched surfaces in the cruise ship were cleaned during this time, particularly inside the cabins, as the housekeepers were not allowed to enter cabins and cleaning supply was provided for passengers to clean their own rooms.(15, 51) In the lack of reliable sources on surface disinfection frequency and efficiency, we simply assumed the

additional surface disinfection efforts reduced the transfer rate of SARS-CoV-2 between fomite and hand by 72.5% ($R_{disinfection}$ in Equation S6) in comparison to a scenario where the cruise ship surfaces were not disinfected during the day, similar to the surface disinfection efficiency assumed in Zhang et al.(12) We assumed that the majority of passengers and crew were not wearing facemasks before the quarantine started, while they all wore facemasks in public areas after the quarantine began. The facemasks were assumed to have an in-vivo filtration protection ($R_{facemask}$) of 95%, similar to protection of surgical masks against SARS-CoV,(52) and a 54% coverage of human facial features ($F_{coverage}$) assuming they only covered mouth and nasal areas.(53) Our approach for deploying these parameters into the mechanistic infection transmission model was described in detail in Sections 1.1 and 1.2.

For the "high efficacy" scenario, we considered the same model parameters for the before quarantine time period; however, we assumed higher efficacy infection control strategies after the quarantine began. In this scenario, we assumed the probability and effectiveness of handwashing, removal efficiency of facemasks, and during-the-day surface viral disinfection efficiency were 99% after the quarantine started. We kept the coverage proportion of the facemasks equal to 54% similar to the previous scenario.

4. Analyzing model outcomes and conducting sensitivity analyses

In this section, we detail how we analyzed model outcomes, defined acceptable model iterations, and conducted a sensitivity analysis to further explore transmission modes and the importance of several key model parameters.

4.1 Selecting acceptable model iterations

The model approach resulted in a total of 21,600 model iterations, with each iteration representing a scenario with distinct combinations of assumptions for unknown or uncertain model input parameters (e.g., incubation period, sub-clinical infectious period, effective reproduction number, viral shedding rates in aerosols and droplets, close-range interaction times), as shown in Table S4. We ran the model with each possible combination of the eight input parameters shown in Table S4 (10×5×6×3×3×2×2×2=21,600) in order to search a wide range of possible parameters and combinations of parameters. With this approach, many of the scenarios are expected to yield unacceptable results because they combine multiple unlikely parameter assumptions (e.g., poor assumptions for incubation period, effective reproduction number, and URT/LRT ratios).

Table S4. Summary of the ranges of 8 unknown or uncertain critical model input parameters that defined each model iteration

**Model Inputs**	Epidemiological Factors			Mechanistic Transmission Factors				
	Effective incubation period	Effective sub-clinical infectious period	Effective reproduction number for the index case	Symptomatic vs asymptomatic emissions	Ratio of aerosol vs. droplet emissions	Minimum close interaction time in cabins	Quarantine infection control efficiency	URT/LRT infectious doses
**No. Scenarios**	10	5	6	2	3	2	2	3
**Range**	6 – 15(28, 29) (days)	1 – 5(33) (days)	1 – 6(23)	0.544(34)<br>1.0(35)	0.3:1(36)<br>2.4:1, 1:1(34)	8 or 12 hours per day*	Moderate(12, 52, 53)<br>High*	1:1<br>10:1<br>100:1*

* Authors' assumptions

For each model iteration, we estimated the number of (1) daily new infected cases and (2) daily cumulative infected cases, and compared estimates to the number of daily and daily cumulative infected cases reported in two sources: Mizumoto et al.(54) for the time span between February 5th and 20th and on the Worldometer website(55) between February 21st and 29th. Worldometer is a reference website that provides counters and real-time statistics for diverse topics including the COVID-19 pandemic. The website's sources of the COVID-19 statistics are official reports, directly from government's communication channels or indirectly, through local media sources when deemed reliable. Worldometer's Covid-19 data is trusted and used by Johns Hopkins CSSE, Financial Times, The New York Times, Business Insider, and many others.(56) The reason for incorporating the Worldometer data is that it accounted for number of infected cases in the cruise ship between February 21st and 24th (i.e., the day that all passengers disembarked) as well as subsequent infection data for those individuals who became infected while aboard the ship prior to showing symptoms, but soon thereafter showed symptoms after leaving the ship.

Figure S4 shows the number of reported infected cases in the Diamond Princess Cruise Ship along with the four considered infection transmission periods, as descried in Section 1.1.

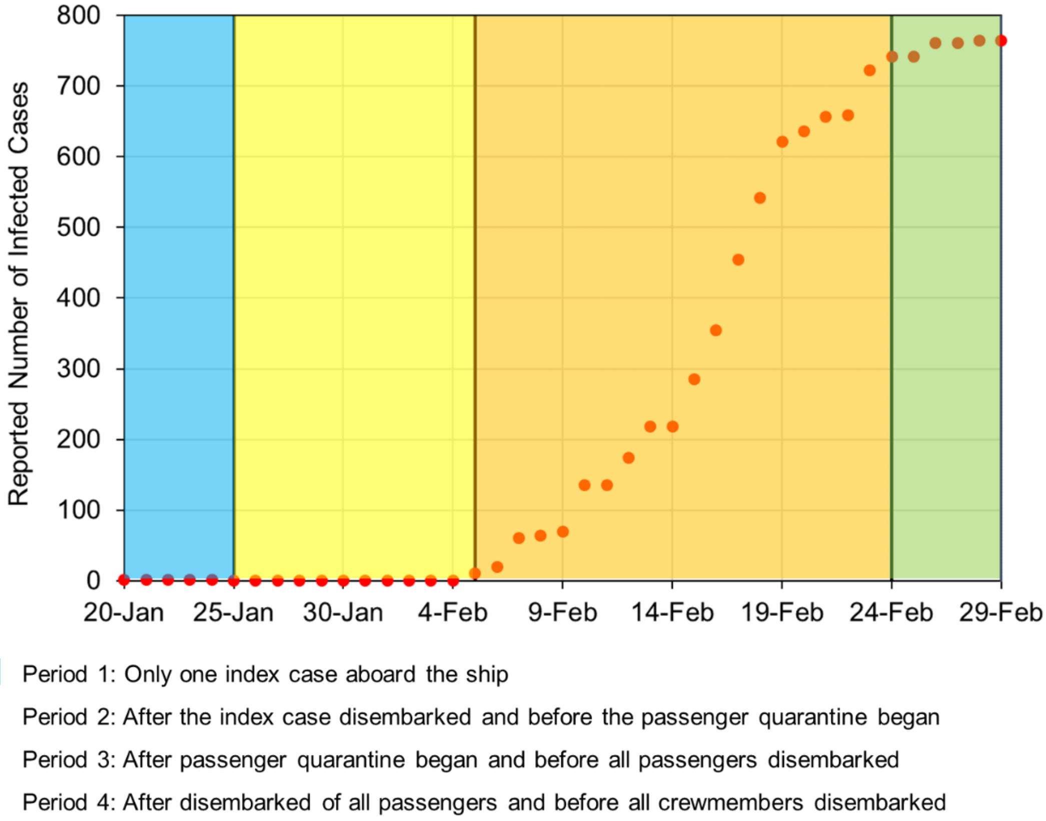

Figure S4. Daily cumulative number of infected cases aboard the Diamond Princess Cruise Ship between January 20, 2020 and February 29, 2020

Coefficients of determination (R^2) were calculated between model predictions and reported case numbers for both daily cases and daily cumulative cases for each of the 21,600 model scenarios. Only those model scenarios that yielded an acceptable coefficient of determination (R^2) between reported and modeled daily and daily cumulative cases were analyzed further to explore the likely ranges and bounds of the unknown or uncertain model input parameters. We considered the

model scenarios with R^2 > 0.95 for daily cumulative cases and a positive R^2 for daily cases as '*acceptable.'* The weaker criterion for daily R^2 values was used because reported daily infection numbers likely suffered from delays in reporting and detection that the model does not predict; however, the cumulative case numbers smooth out these daily variations and warrant more stringent criteria. Results are shown in the main text.

4.2 Model sensitivity to eliminating transmission modes using 'best estimates' of model parameters

In order to explore the importance of each transmission mode, we used a transmission mode elimination process combined with resulting '*best estimates'* of primary model parameters, in which we considered only one transmission mode at a time (e.g., long-range inhalation only, close-range inhalation only, direct deposition, only), as well as combining two transmission modes at a time (e.g., long- and short-range inhalation, direct deposition and fomite).

In this analysis, we assumed the following:

(i) we o*nly* used our 'best estimates' of model parameters,
(ii) we rounded the best estimates of incubation period and subclinical infectious period to integer numbers,
(iii) instead of using the effective reproduction numbers to back-calculate the infectious dose for URT and LRT from the first period of the outbreak simulation for each transmission mode separately, we used the calculated infection doses, calculated using the 'best estimates' of model parameters deployed in the model, and
(iv) the total number of emitted SARS-CoV-2 RNA copies per hour was assumed to be equal to the average of reported emission rates across Liu et al.(36) and Chia et al.(34) (i.e., ~10^6 per hour), with the proportion of emitted inhalable aerosols to the large droplets assumed to be 1.3 (i.e., the 'best estimate' resulting from acceptable model iterations, as shown in Table S5).

Table S5 summarizes the model outcomes when only one or two transmission modes were considered with 'best estimates' of primary model parameters.

Table S5. Summary of infection transmission model results when only one or two transmission modes were considered with 'best estimates' of primary model parameters deployed in the model

Transmission Mode	Daily cumulative R^2	Estimated cases in cabins	Estimated cases in public area	Assumptions / Conditions
All modes	0.981	361	384	'Best estimate' model parameters
Long-range inhalation only	0.130	105	118	No close contact and no hand-to-surface contacts
Close-range inhalation only	0.082	98	118	No hand-to-surface contacts and very high deposition rate
Direct droplet deposition only	-0.467	29	34	No hand-to-surface contact and no inhalable aerosol emission
Fomite only	-0.304	29	69	No close contact and no inhalation rate
Long- & short-range inhalation	0.746	232	247	No hand-to-surface contact
Direct droplet deposition & fomite	-0.285	33	71	No inhalation rate

In all of the cases, COVID-19 infectious doses for URT and LRT was assumed constant and equal to our 'best estimate' back-calculated values.

Results in Table S5 demonstrate the importance of considering all transmission modes, as the R^2 value between predicted and reported daily cumulative case numbers was 0.98 considering all modes. Limiting to any single transmission mode (e.g., only short-range inhalation of aerosols, only long-range inhalation of aerosols, only direct droplet deposition, or only fomite transmission) resulted in R^2 values less than 0.15 and severe undercounting of cases in cabins and public areas. In other words, using our best estimates of several key model parameters, no individual transmission mode can explain reported cases. However, including both long- and short-range inhalation of inhalable aerosols improved model performance ($R^2 = 0.75$), while including direct droplet deposition and fomite together (but ignoring inhalation of aerosols) did not improve model performance. These results suggest that inhalation of smaller diameter aerosols (< 10 µm), across both short-range and long-range distances, were likely the dominant contributor to the transmission of COVID-19 aboard the Diamond Princess Cruise Ship, and that fomite and direct droplet deposition likely played a smaller role.

Table S5 also demonstrates the potential effects of indirectly considered parameters and processes on the estimated contribution of various infection transmission pathways for extreme scenarios. For example, there is some evidence supporting the fact that relative humidity has a critical effect on risk and contribution of different infection transmission pathways (57, 58). We can indirectly test this if we consider a hypothetical extreme scenario, where for example, only large liquid droplets are assumed to carry SARS-CoV-2 because the coronaviruses could lose their viability when droplets reduce to their dry nucleus, the sensitivity analysis shows that the total number of infected cases under this hypothetical direct droplet deposition and fomite only scenario would be only 104 cases (a severe underprediction of cases versus actual cases). As another example, Table S5 shows if we consider another hypothetical extreme scenario, where all SARS-CoV-2 RNA copies were carried by inhalable aerosols smaller than 10 µm in diameter, the number of infected cases on the cruise ship would reduce to 479 cases (a less severe underprediction of cases).

4.3 Model sensitivity to primary epidemiological inputs

Among the 8 unknown or uncertain primary inputs of the developed transmission risk model demonstrated in Table S4, the effective incubation period, effective sub-clinical infectious period, and effective reproduction number for the index case were considered as epidemiological inputs of the model. Table S6 summarizes the coefficients of determination (R^2) for all 21,600 explored model iterations when various combinations of primary epidemiological inputs were adopted in the risk model. Each cell demonstrates the average R^2 value of 72 explored model iterations (i.e. 2 asymptomatic/symptomatic emission scenarios x 3 emission rate scenarios x 2 minimum close interaction time in the cabins scenarios x 3 $ID_{50,URT}/ID_{50,LRT}$ scenarios x 2 infection control efficiency scenarios) combined with a distinct combination of primary epidemic input values used in the risk model.

The vast majority of epidemic model input combinations yielded negative R^2 values, on average, suggesting they were implausible combinations. Table S6 shows several diagonal series of combinations of input values that yielded greater numbers of acceptable iterations, most commonly clustered around effective sub-clinical infection periods of 5 days (with some 2-3 days) and effective incubation periods of 11-13 days.

Table S6. Average coefficients of determination associate with various combinations of primary epidemiological model inputs

**R_{eff} = 1**	C = 6	C = 7	C = 8	C = 9	C = 10	C = 11	C = 12	C = 13	C = 14	C = 15
W = 1	< 0	< 0	< 0	< 0	< 0	< 0	< 0	< 0	< 0	< 0
W = 2	< 0	< 0	< 0	< 0	< 0	< 0	< 0	< 0	< 0	< 0
W = 3	< 0	< 0	< 0	< 0	< 0	< 0	< 0	< 0	< 0	< 0
W = 4	< 0	< 0	< 0	< 0	< 0	< 0	< 0	< 0	< 0	< 0
W = 5	< 0	< 0	< 0	< 0	< 0	< 0	< 0	< 0	< 0	< 0
**R_{eff} = 2**	C = 6	C = 7	C = 8	C = 9	C = 10	C = 11	C = 12	C = 13	C = 14	C = 15
W = 1	0.45	0.18	< 0	< 0	< 0	< 0	< 0	< 0	< 0	< 0
W = 2	0.00	0.70	0.45	< 0	< 0	< 0	< 0	< 0	< 0	< 0
W = 3	< 0	< 0	0.69	0.57	0.03	< 0	< 0	< 0	< 0	< 0
W = 4	< 0	< 0	< 0	0.66	0.62	0.12	< 0	< 0	< 0	< 0
W = 5	< 0	< 0	< 0	< 0	**0.67 (11)**	0.66	0.18	< 0	< 0	< 0
**R_{eff} = 3**	C = 6	C = 7	C = 8	C = 9	C = 10	C = 11	C = 12	C = 13	C = 14	C = 15
W = 1	< 0	0.69	0.55	0.04	< 0	< 0	< 0	< 0	< 0	< 0
W = 2	< 0	< 0	0.18	0.67	0.27	0.20	0.08	< 0	< 0	< 0
W = 3	< 0	< 0	< 0	< 0	0.78	0.44	0.33	0.16	< 0	< 0
W = 4	< 0	< 0	< 0	< 0	< 0	0.85	0.54	0.39	0.18	< 0
W = 5	< 0	< 0	< 0	< 0	< 0	< 0	**0.88 (30)**	0.59	0.39	0.15
**R_{eff} = 4**	C = 6	C = 7	C = 8	C = 9	C = 10	C = 11	C = 12	C = 13	C = 14	C = 15
W = 1	< 0	< 0	0.76	0.49	0.43	0.34	0.18	< 0	< 0	< 0
W = 2	< 0	< 0	< 0	0.66	0.74	0.68	0.53	0.31	0.03	< 0
W = 3	< 0	< 0	< 0	< 0	0.51	**0.88 (21)**	0.79	0.61	0.34	0.03
W = 4	< 0	< 0	< 0	< 0	< 0	0.36	**0.93 (1)**	0.84	0.63	0.31
W = 5	< 0	< 0	< 0	< 0	< 0	0.00	0.26	**0.94 (31)**	0.83	0.58
**R_{eff} = 5**	C = 6	C = 7	C = 8	C = 9	C = 10	C = 11	C = 12	C = 13	C = 14	C = 15
W = 1	< 0	< 0	< 0	0.80	0.78	0.69	0.52	0.24	< 0	< 0
W = 2	< 0	< 0	< 0	< 0	**0.90 (14)**	0.93	0.85	0.65	0.32	< 0
W = 3	< 0	< 0	< 0	< 0	< 0	**0.80 (9)**	0.92	0.89	0.68	0.32
W = 4	< 0	< 0	< 0	< 0	< 0	< 0	0.65	0.88	0.88	0.64
W = 5	< 0	< 0	< 0	< 0	< 0	< 0	< 0	0.55	**0.85 (15)**	0.85
**R_{eff} = 6**	C = 6	C = 7	C = 8	C = 9	C = 10	C = 11	C = 12	C = 13	C = 14	C = 15
W = 1	< 0	< 0	< 0	0.83	0.92	0.90	0.78	0.51	0.18	< 0
W = 2	< 0	< 0	< 0	< 0	0.48	0.74	0.89	0.87	0.59	0.23
W = 3	< 0	< 0	< 0	< 0	< 0	< 0	0.44	0.81	0.88	0.58
W = 4	< 0	< 0	< 0	< 0	< 0	< 0	< 0	0.23	0.78	0.86
W = 5	< 0	< 0	< 0	< 0	< 0	< 0	< 0	< 0	0.19	0.80

* The numbers shown in parenthesizes demonstrate the number of model iterations meeting acceptability criteria (R^2 > 0.95 for daily cumulative cases and a positive R^2 for daily cases)
C: Effective incubation period
W: Effective sub-clinical infectious period
R_{eff}: Effective reproduction number for the index case

4.4 Model sensitivity to changes in the ratio between infectious dose for URT and LRT

The model results demonstrate that the ratio between SARS-CoV-2 infectious dose for upper and lower respiratory tracts plays a critical role in estimating the modes of transmission of COVID-19 aboard the ship. Figures S5 and S6 show the impacts of our three logarithmically spaced assumptions for the ratio between SARS-CoV-2 infectious doses for URT and LRT on the estimates of infection contribution of various transmission modes and viral sources among the 132 acceptable model iterations.

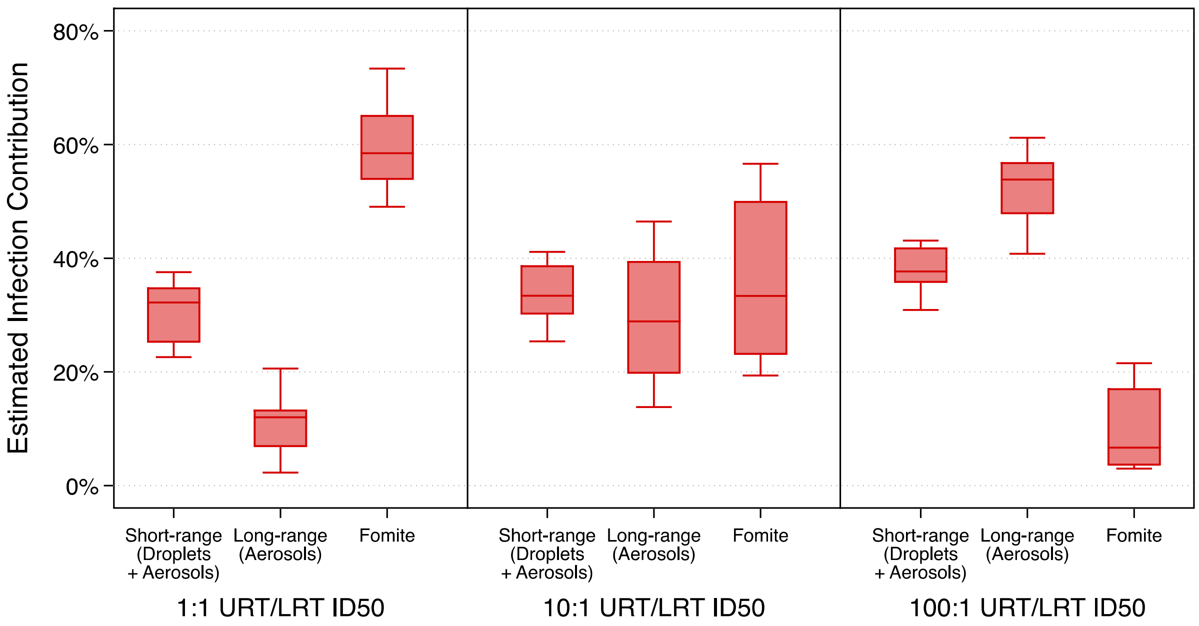

Figure S5. Estimated infection contributions of various transmission modes corresponding to different $ID_{50,URT}$ / $ID_{50,LRT}$ scenarios among acceptable model iterations

Figure S5 demonstrates that when the SARS-CoV-2 infectious doses for URT and LRT are assumed to be equal (1:1 URT/LRT ID_{50}), the contribution of fomites to infection transmission in the cruise ship was significantly higher than the other transmission modes (i.e., long- and short-range). This means that ~60% of the total number of infectious SARS-CoV-2 that reached to the respiratory tracts of susceptible individuals was estimated to reach to URT via fomite pathways. However, when we considered a lower infectious dose for LRT of susceptible individuals, our estimates of contribution of long- and short-range transmission modes in number of infected cases was increased. For an assumption of URT infectious dose 10 times higher than the LRT infectious dose, the estimated infection contributions of all transmission modes were approximately similar. For an assumption of URT infectious dose 100 times higher than the LRT infectious dose, more than 90% of infection transmission was estimated to be via short- and long-range transmission modes. Clearly, this ratio is a critical factor in the model and remains to be better understood from clinical investigations. However, recall that a ratio of 100:1 had the largest number of acceptable model iterations associated with the assumption (i.e., 58 compared to 35 for 1:1 and 39 for 10:1), with an average ratio of ~47:1 (Table 2 in the main text), which provides some guidance on where this value may reasonably lie.

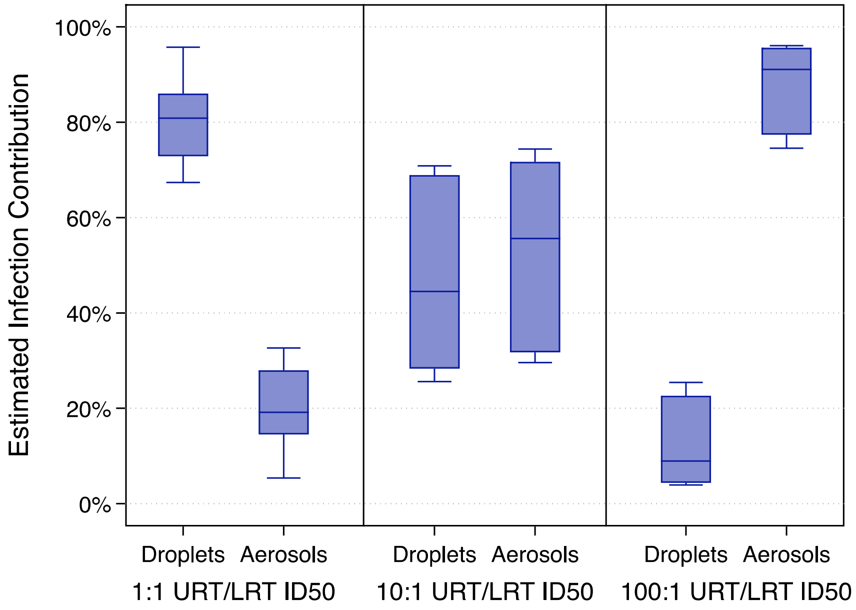

Figure S6. Estimated infection contributions of viral transmission sources corresponding to different $ID_{50,URT}$ / $ID_{50,LRT}$ scenarios among acceptable model iterations

Similarly, our assumptions for the ratio between infectious dose for URT and LRT affected our estimates of infection contribution of different viral sources. In the 1:1 URT/LRT ID_{50} scenario, transmission trough larger droplets (> ~ 10 µm in diameter) was ~ 4 times higher than the transmission through aerosols. Conversely, in the 100:1 URT/LRT ID_{50} scenario, the infection transmission via smaller inhalable aerosols (< ~ 10 µm in diameter) was ~9 times higher than of large droplets. The infection contributions of droplets and aerosols were approximately similar for the 10:1 URT/LRT ID_{50} scenario. One thing to notice is because we assumed inhalable aerosols and large droplets deposit on LRT and URT, respectively, the infection contribution of aerosols and droplets could also be considered as the infection contribution of LRT and URT, respectively.

4.5 Model sensitivity to changes in emission rate scenarios

Finally, we explored the sensitivity of our model results to the changes in our assumptions for the emission rates of SARS-CoV-2 in the form of droplets and aerosols. Figures S7 and S8 demonstrate the impacts of ratio of aerosol versus droplet emissions on estimated infection contributions of various transmission modes and viral sources, respectively.

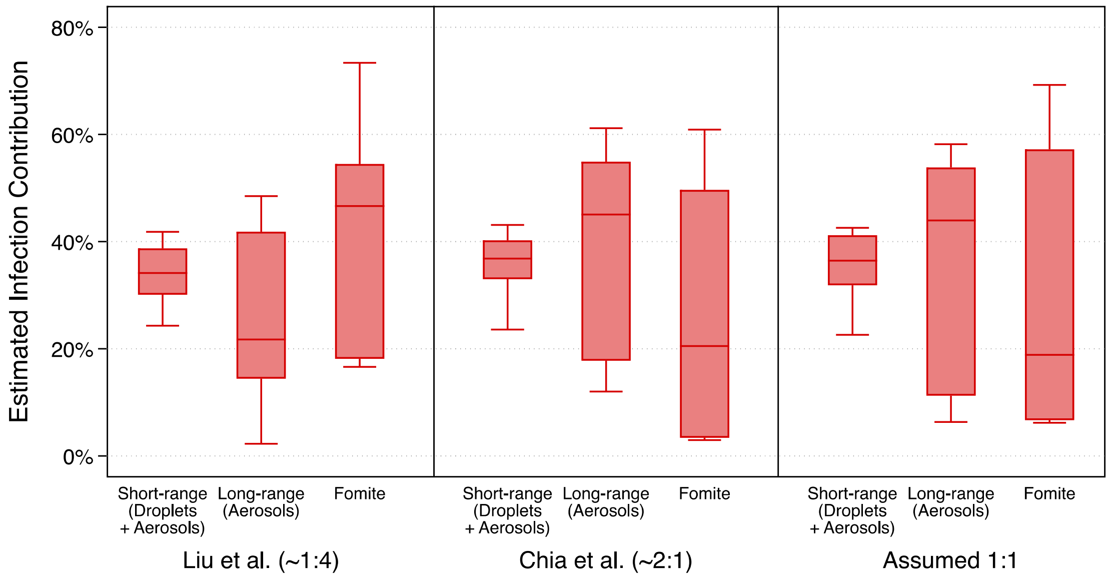

Figure S7. Estimated infection contributions of various transmission modes corresponding to three different assumptions for the ratio between aerosol and droplet emissions among acceptable model iterations (i.e., aerosol/droplet ratios of ~1:4, ~2:1, and 1:1)

Figure S7 shows that the median estimated infection contribution from fomite transmission mode is ~50%, and is higher than both short- and long- range transmission modes, when we assumed that the ratio of SARS-CoV-2 emitted in the form of droplets is about 4 times higher than the number of viruses emitted in inhalable aerosols (based on approximations from data reported in Liu et al.(36)). The median estimated infection contribution of long-range transmission during this emission scenario was only ~20%, but then increased to ~45% when the ratio of aerosol versus droplet emissions increased to 2:1 (based on approximations from data reported in Chia et al.(34)). The estimated infection contribution of short-range transmission, which was a combination of deposition of larger droplets on URT and inhalable aerosols on LRT both within close-contact range, were approximately similar for all three emission ratio scenarios. When we assumed a similar emission rate of SARS-CoV-2 in forms of droplet and aerosols (i.e., 1:1), the median infection contribution of short-range, long-range, and fomite transmission modes were estimated to be ~35%, ~45%, and ~20%, respectively. This remains an uncertain parameter, with approximately equal numbers of acceptable model iterations associated with each assumption (i.e., Table 2 in the main text).

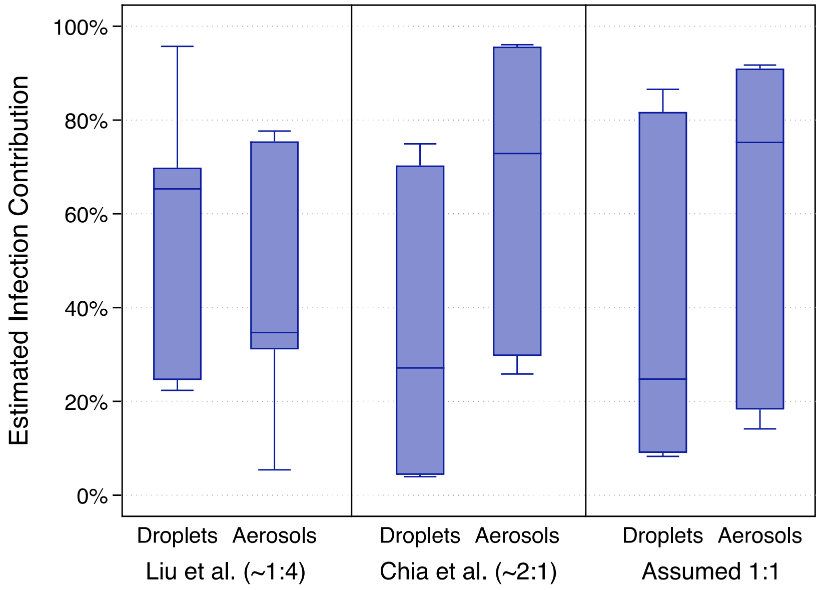

Figure S8. Estimated infection contributions of viral transmission sources corresponding to three different assumptions for the ratio between aerosol and droplet emissions among acceptable model iterations (i.e., aerosol/droplet ratios of ~1:4, ~2:1, and 1:1)

Similarly, Figure S8 shows the infection contribution via droplets was higher using estimates of aerosol/droplet ratios based on Liu et al.(36) (~1:4), with a median value of ~65%, and it decreased to ~30% when we assumed aerosol/droplet ratios of ~2:1 based on Chia et al.(34) One thing to notice is the large variation in the estimated infection contributions based on the emission scenarios observed in Figures S7 and S8 is due primarily to changes in our assumptions for the infectious doses of upper and lower respiratory tracts, as explained in Section 4.4.

4.6 Statistical significance testing on the model results

We performed a Mann-Whitney U-test on the model results to evaluate the statistical significance of each mode comparison. For the main analysis, Table S7 indicates that the estimated infection contributions by droplets and aerosols, as well as between all transmission modes, are significantly different after the passenger quarantine started ($p < 0.0001$). However, differences in the estimated infection contributions between droplets and aerosols, and between short-range and fomite transmission, before isolation started, and between long-range and both short-range and fomite transmission modes for the entire modeling duration are not statistically different ($p > 0.05$).

Table S7. Mann-Whitney U-test results on statistical significance difference between estimated infection contributions of various viral sources (droplet and aerosols) and transmission modes (short-range, long-range, and fomite)

Comparison category	Comparison sub-category	P-value		
		Entire duration	Before isolation	After isolation
Viral source	Droplets – Aerosols	< 0.0001	0.3175	< 0.0001
Transmission mode	Short-range – Long-range	0.2157	< 0.0001	< 0.0001
Transmission mode	Short-range – Fomite	< 0.0001	0.5675	< 0.0001
Transmission mode	Long-range – Fomite	0.1701	0.0004	< 0.0001

Table S8 shows the results of Mann-Whitney U-tests comparing the infection contributions of various viral sources and transmission modes when different assumptions for several mechanistic transmission factors are adopted for the entire modeling duration. The estimated infection contributions of various viral sources and transmission modes when different URT/LRT infectious dose scenarios are adopted are statistically different with p-values lower than ~0.01. They also show that the adopted scenarios for symptomatic versus asymptomatic emissions do not yield a significant difference in the estimates of infection contributions. The statistical significance testing on estimates of infection contributions of various viral sources and transmission modes when different mechanistic transmission factor scenarios are adopted demonstrates a mixed result as shown in Table S8. For example, it is shown that infection contribution of short-range transmission mode is statistically different when the minimum close interaction time in cabins was assumed to be 8 hours or 16 hours ($p < 0.0001$), but the infection contribution of droplets with similar assumptions is not significantly different.

Table S8. Mann-Whitney U-test results on statistical significance difference between estimated infection contributions of various viral sources (droplet and aerosols) and transmission modes (short-range, long-range, and fomite) in the entire modeling duration when different mechanistic transmission factors are adopted

Mechanistic Transmission Factors	P-value*		
	**Comparison Scenario I**	**Comparison Scenario II**	**Comparison Scenario III**
Symptomatic vs asymptomatic emission scenarios	0.544 – 1.0	N/A	N/A
Viral source – Droplets	0.1330	N/A	N/A
Viral source – Aerosols	0.1283	N/A	N/A
Transmission mode – Long-range	0.1216	N/A	N/A
Transmission mode – Short-range	0.7089	N/A	N/A
Transmission mode – Fomite	0.1822	N/A	N/A
Ratio of aerosol vs. droplet emission scenarios	0.3:1 – 2.4:1	0.3:1 – 1:1	2.4:1 – 1:1
Viral source – Droplets	0.0371	0.0105	0.0272
Viral source – Aerosols	0.0408	0.0105	0.0272
Transmission mode – Long-range	0.0030	0.0105	0.1675
Transmission mode – Short-range	0.0599	0.1127	0.6435
Transmission mode – Fomite	0.0088	0.0185	0.0355
Minimum close interaction time in cabin scenarios	8 hrs – 16 hrs	N/A	N/A
Viral source – Droplets	0.5754	N/A	N/A
Viral source – Aerosols	0.5238	N/A	N/A
Transmission mode – Long-range	0.0094	N/A	N/A
Transmission mode – Short-range	< 0.0001	N/A	N/A
Transmission mode – Fomite	0.5179	N/A	N/A
Quarantine infection control efficiency scenarios	Moderate – High	N/A	N/A
Viral source – Droplets	0.0468	N/A	N/A
Viral source – Aerosols	0.0555	N/A	N/A
Transmission mode – Long-range	0.2679	N/A	N/A
Transmission mode – Short-range	0.0107	N/A	N/A
Transmission mode – Fomite	0.0296	N/A	N/A
URT/LRT infectious dose scenarios	1:1 – 10:1	1:1 – 100:1	10:1 – 100:1
Viral source – Droplets	< 0.0001	< 0.0001	< 0.0001
Viral source – Aerosols	< 0.0001	< 0.0001	< 0.0001
Transmission mode – Long-range	< 0.0001	< 0.0001	< 0.0001
Transmission mode – Short-range	0.0165	< 0.0001	< 0.0001
Transmission mode – Fomite	< 0.0001	< 0.0001	< 0.0001

* The compared estimated infection contributions are not significantly different at any level smaller than the calculated p-values

References

1. M. Nicas, R. M. Jones, Relative contributions of four exposure pathways to influenza infection risk. *Risk Analysis* **29**, 1292–1303 (2009).

2. M. Nicas, G. Sun, An integrated model of infection risk in a health-care environment. *Risk Anal.* **26**, 1085–1096 (2006).

3. R. M. Jones, *et al.*, Characterizing the risk of infection from Mycobacterium tuberculosis in commercial passenger aircraft using quantitative microbial risk assessment. *Risk Analysis* **29**, 355–365 (2009).

4. R. M. Jones, E. Adida, Influenza infection risk and predominate exposure route: uncertainty analysis. *Risk Analysis* **31**, 1622–1631 (2011).

5. R. M. Jones, M. Nicas, Benchmarking of a Markov Multizone Model of Contaminant Transport. *Ann Occup Hyg* **58**, 1018–1031 (2014).

6. R. M. Jones, M. Nicas, Experimental evaluation of a Markov multizone model of particulate contaminant transport. *Ann Occup Hyg* **58**, 1032–1045 (2014).

7. L. F. Moriarty, Public Health Responses to COVID-19 Outbreaks on Cruise Ships—Worldwide, February–March 2020. *MMWR. Morbidity and Mortality Weekly Report* **69** (2020).

8. Princess Cruises, Princess Cruises: Diamond Princess - Cruise Ship Information. *www.princess.com* (2020) (April 2, 2020).

9. CDC, Guidance for Cruise Ships on Influenza-like Illness (ILI) Management | Quarantine | CDC (2019) (March 19, 2020).

10. CDC, “Centers for Disease Control and Prevention - Alert letter for. Diamond Princess Passengers and Crew Members” (2020).

11. L. Zheng, Q. Chen, J. Xu, F. Wu, Evaluation of intervention measures for respiratory disease transmission on cruise ships. *Indoor and Built Environment* **25**, 1267–1278 (2016).

12. N. Zhang, R. Miao, H. Huang, E. Y. Chan, Contact infection of infectious disease onboard a cruise ship. *Scientific reports* **6**, 38790 (2016).

13. R. Vivancos, *et al.*, Norovirus outbreak in a cruise ship sailing around the British Isles: Investigation and multi-agency management of an international outbreak. *Journal of Infection* **60**, 478–485 (2010).

14. J. Moya, *et al.*, Exposure factors handbook: 2011 edition. *US Environmental Protection Agency* (2011).

15. Princess Plus, Princess Cruises: Diamond Princess Updates - Notices & Advisories. *www.princess.com* (2020) (April 2, 2020).

16. N. van Doremalen, *et al.*, Aerosol and Surface Stability of SARS-CoV-2 as Compared with SARS-CoV-1. *New England Journal of Medicine* (2020).

17. S. Xiao, Y. Li, M. Sung, J. Wei, Z. Yang, A study of the probable transmission routes of MERS-CoV during the first hospital outbreak in the Republic of Korea. *Indoor Air* **28**, 51–63 (2018).

18. H. Lei, *et al.*, Routes of transmission of influenza A H1N1, SARS CoV, and norovirus in air cabin: Comparative analyses. *Indoor Air* **28**, 394–403 (2018).

19. R. M. Jones, Critical Review and Uncertainty Analysis of Factors Influencing Influenza Transmission. *Risk Analysis* **31**, 1226–1242 (2011).

20. M. Nicas, D. Best, A study quantifying the hand-to-face contact rate and its potential application to predicting respiratory tract infection. *Journal of occupational and environmental hygiene* **5**, 347–352 (2008).

21. P. Göker, M. G. Bozkir, Determination Of Hand And Palm Surface Areas As A Percentage Of Body Surface Area In Turkish Young Adults. *Trauma and Emergency Care* **2**, 1–4 (2017).

22. W. Chen, N. Zhang, J. Wei, H.-L. Yen, Y. Li, Short-range airborne route dominates exposure of respiratory infection during close contact. *Building and Environment* **176**, 106859 (2020).

23. K. Mizumoto, G. Chowell, Transmission potential of the novel coronavirus (COVID-19) onboard the diamond Princess Cruises Ship, 2020. *Infectious Disease Modelling* **5**, 264–270 (2020).

24. H. Wahlström, *et al.*, A Reed-Frost model of the spread of tuberculosis within seven Swedish extensive farmed fallow deer herds. *Preventive Veterinary Medicine* **35**, 181–193 (1998).

25. R. T. Chen, G. M. Goldbaum, S. G. F. Wassilak, L. E. Markowitz, W. A. Orenstein, AN EXPLOSIVE POINT-SOURCE MEASLES OUTBREAK IN A HIGHLY VACCINATED POPULATIONMODES OF TRANSMISSION AND RISK FACTORS FOR DISEASE. *Am J Epidemiol* **129**, 173–182 (1989).

26. H. Abbey, An examination of the Reed-Frost theory of epidemics. *Human biology* **24**, 201 (1952).

27. , Coronavirus Update: Diamond Princess Passengers Leave Ship As Expert Slams Quarantine. *NPR.org* (June 6, 2020).

28. S. A. Lauer, *et al.*, The Incubation Period of Coronavirus Disease 2019 (COVID-19) From Publicly Reported Confirmed Cases: Estimation and Application. *Annals of Internal Medicine* **172**, 577–582 (2020).

29. Q. Li, *et al.*, Early Transmission Dynamics in Wuhan, China, of Novel Coronavirus–Infected Pneumonia. *New England Journal of Medicine* (2020) https:/doi.org/10.1056/NEJMoa2001316 (May 23, 2020).

30. J. Arzt, *et al.*, Quantitative impacts of incubation phase transmission of foot-and-mouth disease virus. *Scientific Reports* **9**, 1–13 (2019).

31. H. A. Rothan, S. N. Byrareddy, The epidemiology and pathogenesis of coronavirus disease (COVID-19) outbreak. *Journal of Autoimmunity* **109**, 102433 (2020).

32. J. Hellewell, *et al.*, Feasibility of controlling COVID-19 outbreaks by isolation of cases and contacts. *The Lancet Global Health* **8**, e488–e496 (2020).

33. R. Huang, J. Xia, Y. Chen, C. Shan, C. Wu, A family cluster of SARS-CoV-2 infection involving 11 patients in Nanjing, China. *The Lancet Infectious Diseases* **20**, 534–535 (2020).

34. P. Y. Chia, *et al.*, Detection of air and surface contamination by SARS-CoV-2 in hospital rooms of infected patients. *Nature Communications* **11**, 2800 (2020).

35. M. M. Arons, *et al.*, Presymptomatic SARS-CoV-2 Infections and Transmission in a Skilled Nursing Facility. *New England Journal of Medicine* **0**, null (2020).

36. Y. Liu, *et al.*, Aerodynamic analysis of SARS-CoV-2 in two Wuhan hospitals. *Nature*, 1–4 (2020).

37. S. Tone, Inside Wuhan's 'Fangcang' Shelter Hospitals. *Sixth Tone* (2020) (March 31, 2020).

38. , Wuhan to convert gymnasium, exhibition center into temporary hospitals - Xinhua | English.news.cn (May 25, 2020).

39. C. Yang, P. Demokritou, Q. Chen, J. Spengler, A. Parsons, Ventilation and air quality in indoor ice skating arenas. *ASHRAE Transactions* **106**, 338 (2000).

40. W. J. Riley, T. E. McKone, A. C. K. Lai, W. W. Nazaroff, Indoor Particulate Matter of Outdoor Origin: Importance of Size-Dependent Removal Mechanisms. *Environ. Sci. Technol.* **36**, 200–207 (2002).

41. P. Azimi, B. Stephens, HVAC filtration for controlling infectious airborne disease transmission in indoor environments: Predicting risk reductions and operational costs. *Building and Environment* **70**, 150–160 (2013).

42. D. K. Milton, M. P. Fabian, B. J. Cowling, M. L. Grantham, J. J. McDevitt, Influenza Virus Aerosols in Human Exhaled Breath: Particle Size, Culturability, and Effect of Surgical Masks. *PLOS Pathogens* **9**, e1003205 (2013).

43. J. Yan, *et al.*, Infectious virus in exhaled breath of symptomatic seasonal influenza cases from a college community. *PNAS* **115**, 1081–1086 (2018).

44. U.S. Department of Homeland Security Science and Technology, "Master Question List for COVID-19 (caused by SARS-CoV-2): Weekly Report" (Hazard Awareness & Characterization Technology Center, 2020).

45. T.-C. Hsiao, H.-C. Chuang, S. M. Griffith, S.-J. Chen, L.-H. Young, COVID-19: An Aerosol's Point of View from Expiration to Transmission to Viral-mechanism. *Aerosol Air Qual. Res.* **20**, 905–910 (2020).

46. R. J. Thomas, Particle size and pathogenicity in the respiratory tract. *Virulence* **4**, 847–858 (2013).

47. P. Azimi, *Advancing knowledge of indoor aerosol sources, fate, transport, and control* (Illinois Institute of Technology, 2016).

48. R. H. Alford, J. A. Kasel, P. J. Gerone, V. Knight, Human Influenza Resulting from Aerosol Inhalation. *Proceedings of the Society for Experimental Biology and Medicine* **122**, 800–804 (1966).

49. B. Bean, *et al.*, Survival of Influenza Viruses on Environmental Surfaces. *J Infect Dis* **146**, 47–51 (1982).

50. K. Kakimoto, *et al.*, Initial investigation of transmission of COVID-19 among crew members during quarantine of a cruise ship—Yokohama, Japan, February 2020 (2020).

51. , 27 Days in Tokyo Bay: What Happened on the Diamond Princess. *Wired* (June 25, 2020).

52. Y. Li, *et al.*, In vivo protective performance of N95 respirator and surgical facemask. *American Journal of Industrial Medicine* **49**, 1056–1065 (2006).

53. W. Chen, N. Zhang, J. Wei, H.-L. Yen, Y. Li, Short-range airborne route dominates exposure of respiratory infection during close contact. *medRxiv*, 2020.03.16.20037291 (2020).

54. K. Mizumoto, K. Kagaya, A. Zarebski, G. Chowell, Estimating the asymptomatic proportion of coronavirus disease 2019 (COVID-19) cases on board the Diamond Princess cruise ship, Yokohama, Japan, 2020. *Eurosurveillance* **25**, 2000180 (2020).

55. Worldometer, February 2020 Coronavirus News Updates - Worldometer (2020) (April 3, 2020).

56. , Worldometer - FAQ (June 8, 2020).

57. W. Yang, L. C. Marr, Mechanisms by which ambient humidity may affect viruses in aerosols. *Applied and environmental microbiology* **78**, 6781–6788 (2012).

58. M. D. Sobsey, J. S. Meschke, Virus survival in the environment with special attention to survival in sewage droplets and other environmental media of fecal or respiratory origin. *Report for the World Health Organization, Geneva, Switzerland* **70** (2003).
